# Life cycle adapted upstream open reading frames (uORFs) in *Trypanosoma congolense*: A post-transcriptional approach to accurate gene regulation

**DOI:** 10.1371/journal.pone.0201461

**Published:** 2018-08-09

**Authors:** Philipp Fervers, Florian Fervers, Wojciech Makałowski, Marcin Jąkalski

**Affiliations:** 1 University of Münster, Faculty of Medicine, Institute of Bioinformatics, Münster, Germany; 2 Karlsruhe Institute of Technology, Department of Informatics, Karlsruhe, Germany; National Center for Biotechnology Information, UNITED STATES

## Abstract

The presented work explores the regulatory influence of upstream open reading frames (uORFs) on gene expression in *Trypanosoma congolense*. More than 31,000 uORFs in total were identified and characterized here. We found evidence for the uORFs’ appearance in the transcriptome to be correlated with proteomic expression data, clearly indicating their repressive potential in *T*. *congolense*, which has to rely on post-transcriptional gene expression regulation due to its unique genomic organization. Our data show that uORF’s translation repressive potential does not only correlate with elemental sequence features such as length, position and quantity, but involves more subtle components, in particular the codon and amino acid profiles. This corresponds with the popular mechanistic model of a ribosome shedding initiation factors during the translation of a uORF, which can prevent reinitiation at the downstream start codon of the actual protein-coding sequence, due to the former extensive consumption of crucial translation components. We suggest that uORFs with uncommon codon and amino acid usage can slow down the translation elongation process in *T*. *congolense*, systematically deplete the limited factors, and restrict downstream reinitiation, setting up a bottleneck for subsequent translation of the protein-coding sequence. Additionally we conclude that uORFs dynamically influence the *T*. *congolense* life cycle. We found evidence that transition to epimastigote form could be supported by gain of uORFs due to alternative trans-splicing, which down-regulate housekeeping genes’ expression and render the trypanosome in a metabolically reduced state of endurance.

## Introduction

Trypanosomes are flagellate, unicellular parasites, belonging to the class of *Kinetoplastida* [1]. *T*. *congolense* is the infective agent of the animal African trypanosomiasis (AAT) and probably the most widespread pathogen of livestock in the sub-Saharan Africa [2]. It is transmitted during the blood meal of tsetse fly, which confronts the trypanosome with unique environmental constraints of two different hosts. Four consecutive life cycle stages, namely bloodstream (BSF), procyclic (PCF), epimastigote (EMF), and metacyclic (MCF) assure adaptation to the changing environment of the parasite.

In contrast to higher eukaryotes, the genome of kinetoplastids is organized in polycistronic transcription units (PTUs), each consisting of approximately 10 to 100 protein-coding genes. Such gene clusters generally comprise of functionally unrelated genes [3–6]. RNA polymerase II binds to the boundaries of adjacent PTUs and transcribes them as long precursor mRNAs. Virtually no promoters are involved in the transcription process [7]. Subsequently, trypanosome transcripts undergo processing of their ends in order to generate mature mRNAs–here a 39 nt-long spliced leader (SL) is trans-spliced to the 5' end of a nascent mRNA (splice acceptor site, SAS) and the 3’ end is polyadenylated co-transcriptionally [4]. By employing different SASs, alternative trans-splicing results in longer 5’ of a given gene and a respective shorter 3’ UTR of its upstream neighbor, or *vice versa*, keeping a near constant distance between both ends. The alternative trans-splicing has been shown to be wide-spread in *T*. *brucei* and hypothesized to contribute to its stage specific gene regulation by gain or loss of UTR-embedded sequence features [8].

The organization of *T*. *congolense’s* genome in PTUs doesn’t allow control of transcription initiation at the level of individual genes [4]. PTUs are transcribed as a whole, which implies a simplified model that every processed monocistronic mRNA arising from the same PTU should appear with the same abundance in the cytosol. Gene expression levels are then principally tweaked post-transcriptionally [3,4,9], which is reflected by the weak correlation between mRNA and protein levels [10]. The amount of peptide translated from each transcript is determined by two factors: the mRNA’s stability, thus lifespan, and the translation efficiency, which limits the amount of peptide synthesized per time. Different processes have been suggested to influence mRNA stability and translation efficiency, ultimately regulating peptide expression: association of RNA-binding proteins (RBPs) with 3’ UTR can increase or decrease the transcripts expression, interaction with cap binding factors can influence ribosome recruitment and variation of mRNA digestion processes controls mRNA life [4]. Additionally, codon usage has been proposed to act upon translation efficiency and to be a major point of action for gene regulation in trypanosomes [11]. In this work, *T*. *congolense* is employed as a model organism to investigate gene regulation by upstream open reading frames (uORFs), as it allows to conduct such research among all four major life cycle stages of trypanosome [12,13]. The regulative effect of uORFs in trypanosomes has already been suggested for *T*. *brucei* [10,14,15].

uORFs are defined as usually small open reading frames, which are located within the 5’ untranslated region of a mature mRNA. Their initiation codon lies in the 5’ UTR, but the in-frame stop codon can be located upstream or sometimes even downstream of the start codon of the primary CDS [16]. Specifically, with regard to trypanosomes, uORFs are located between the trans-splicing sites (spliced leader acceptor sites) and the start codon of CDS within a mature mRNA. In general, the ribosome’s small subunit (SSU) recruits the large subunit (LSU) on the first start codon it encounters (first-AUG rule, Kozak [17]). In this basic concept, regardless if the most upstream AUG opens a CDS of a gene or rather a uORF, translation initiation will take place at this first start codon. Introduction of reporter genes into uORFs, mass spectrometry experiments [17,18] and recent ribosome profiling experiment in *T*. *brucei* [14] have proven translational activity at uORFs. In case the ribosome doesn’t fully disassociate from the mRNA upon termination of uORF translation, but the SSU continues scanning towards the 5’ end (“partial dissociation”), the ribosome can reinitiate at a downstream AUG for translation of another ORF [17,19,20]. In fact, less than 50% of ribosomes, which translate a uORF, indeed undergo reinitiation at a downstream AUG as shown for mammals [19]. Several structural features of a uORF and of its context in the 5’ UTR influence the chances for the event of downstream reinitiation to take place. The uORF’s position in the 5’ UTR crucially defines the likelihood of reinitiation at downstream AUG by setting up a time restriction for re-acquisition of Met-tRNA_i_ during the migration of the SSU from uORF stop to CDS start [17]. Met-tRNA_i_ is obligatory for recognition and pairing of the start codon. A longer distance from uORF stop codon to the downstream CDS start codon facilitates reinitiation, whereas a short uORF to CDS distance can prevent the SSU from reinitiation [21]. The bottleneck is set up by activity of eIF2, which catalyzes the binding of Met-tRNA_i_ to the SSU [17,22]. eIF2 has been proven a central factor to regulate protein synthesis in trypanosomes [23], as in general for other eukaryotes. Generally, the repressive effect of a uORF is restricted to a uORF to CDS distance window of less than 80 nt [24,25]. For higher uORF to CDS distances, the time window for reacquisition of Met-tRNA_i_ is above threshold, and vi*ce versa C*DS overlapping uORFs can shunt ribosomes over the CDS’s AUG, unconditionally rendering the uORF translating ribosomes unavailable for CDS translation [17,24]. Furthermore, a uORF’s sequence length might influence its repressive potential by defining the duration of the elongation process, which refers to a mechanical model of initiation factors, i.e. eIF4G and eIF3 being dynamically shed during elongation [17,26]. This concept should also apply for kinetoplastids, since particularly the eIF3 is largely conserved between e.g. mammals and *Leishmania* [27]. More comprehensive description of translation repression defining uORF characteristics has been provided recently [26].

The nucleotide sequence of a uORF is not considered to have a major impact on its translation repressive potential [19]. However, regarding the translation of CDSs, usage of a species-specific optimum set of codons has been shown to increase translation elongation speed and accuracy [28]. *Vice versa*, the usage of inefficient codons in a CDS slows down the elongation process. Transferring this concept to uORFs, inefficient codon usage extends the time a ribosome spends in elongation phase while translating a uORF, which decreases reinitiation efficiency [26]. Recently, usage of inefficient codons in uORFs to be a negative factor for mRNA stability has been suggested [29], as already previously demonstrated for CDSs [28].

uORFs dynamically repress downstream translation in a dose dependent manner [21], meaning the more uORFs are present in a 5’ UTR and the more unfavorable features they possess, the less likely chances for reinitiation are. Brow et al. [30] have shown different translation efficiencies for human oncogene transcript variants, containing a different number of uORFs due to alternating promoter usage. A translation regulative nature has been assumed for uORFs in trypanosomes as well [14]. As a result of alternative trans-splicing and thus alternating 5’ UTR lengths of individual genes, transcript variants in *T*. *congolense* can contain a variable number of uORFs, as already shown in *T*. *brucei* [8]. The presented work investigates, if alternative trans-splicing in *T*. *congolense* affects the presence of identified uORFs in the stage-specific transcriptome and ultimately influences gene expression throughout the life cycle. Furthermore, the impact of uORF codon and amino acid usage on reinitiation efficiency is explored.

## Materials and methods

### Genomic dataset

Genomic data of *T*. *congolense* [31], gene annotations and nucleotide sequences were obtained from the TriTrypDB database (www.tritrypdb.org, release 25, last access date 15.06.2016 [32]). Annotations of 5' UTRs, which were not available in TriTrypDB at the time of the study, were derived from the *T*. *congolense* resequencing and reannotation project (see S6 Data, Jąkalski et al. unpublished). Briefly, all raw reads from RNA-seq experiment (Illumina) including samples representing all four major life cycle stages of *T*. *congolense* were screened for presence of a fragment of the 39 nt-long spliced leader (SL) sequence [4]. Such reads were then trimmed of the SL motif (plus its upstream sequence) and mapped back to the *T*. *congolense* genome (TriTrypDB 25), thus defining the exact locations of trans-splicing events. Subsequently, the identified sites were linked to the nearest downstream protein-coding genes (as annotated in TriTrypDB), hence providing information on 5’ untranslated regions for these genes. This way, 5’ UTRs were annotated for 8,345 out of 13,148 protein-coding genes annotated in TriTrypDB 25. Annotations of 5' gene ends comprise information on alternative sites within (more than one site per gene) and between all four life cycle stages of the parasite (gain/loss of trans-splicing site in different stages). Each such variant of single trans-splicing site linked to its downstream protein-coding gene (i.e. 5’ UTR + CDS) was considered as individual mature mRNA in our analyses. Proteomic expression data (relative protein expression changes between the life cycle stages) of *T*. *congolense* [12] were obtained from the TriTrypDB resources. Gene expression profiles derive from the high throughput transcriptome sequencing (Illumina RNA-seq) experiments of all four life cycle stages of *T*. *congolense* (see S7 Data, Jąkalski et al. unpublished), in which the sequenced reads were mapped to the genes annotated in TriTrypDB 25 to estimate their mRNA abundance levels.

### Identification of uORFs

Each start codon in 5’ UTR followed by an in-frame stop codon is to be considered a uORF. There is no minimum length, because the scanning ribosome doesn’t recognize the dimension of the uORF in advance before assembling [21]. This approach has already been adopted e.g. for *T*. *brucei* [14]. To assign a unique name to each uORF, the stop codons with at least one in-frame start codon in the 5’ UTR were counted. To each stop codon the respective start codon(s) were linked. These two parameters of a uORF in combination with the gene’s CDS make up a unique name (see S1 Fig). uORFs can often be partially redundant, when several start codons in the same frame are followed by the same stop codon. If not stated otherwise, in the presented analyses only the longest uORF variant for each stop codon and the observed stage was used. To avoid restricting uORF identification to the ideal concept of only the first AUG being the start of translation, uORF IDs were assigned to all open reading frames in 5’ UTR, disregarding their status as the longest variant (S1 Fig).

### Codon Adaptation Index (CAI)

The codon adaptation index (CAI) was first introduced by Sharp and Li [33] and is a measure for the similarity of codon bias in a CDS to a reference group of extensively expressed CDSs, which require excellent translation efficiency and accuracy. The presented work uses the algorithm for CAI calculation published by Carbone et al. [34].

### Resemblance of amino acid profiles–Amino Acid Similarity Index (ASI)

No convenient method for comparing the amino acid profile of a given ORF to a background set of ORFs has been described in the literature so far. There exists a preference to study frequencies of individual amino acids or exclusively focus on codon usage. In order to achieve a figure for a given ORF, which represents the amino acid usage similarity to a background set of ORFs, the Amino Acid Similarity Index (ASI) was established here. Each amino acid *A* is used by a mean frequency *f*_*mA*_ throughout the set of ORFs. *f*_*oA*_ is the frequency of the respective amino acid in the currently observed ORF. The difference *f*_*mA*_*—f*_*oA*_ is a measure for the resemblance of the individual ORF's frequency of the amino acid compared to the background set of ORFs. Adapting the least square concept, which is employed in regression analysis to calculate an optimum line, minimizing the overall distance from a group of dots, the difference *f*_*mA*_*—f*_*oA*_ is squared. Summing up the squared differences for all twenty amino acids and the stop codon results in the ASI, similar to the sum of distance squares in fitting a regression line.

$$ASI=\sum {\left(f\mathrm{mA}-f\mathrm{oA}\right)}^{2}$$

CAI and ASI perform best when analyzing long sequences. Throughout shorter sequences, the influence of single elements can be overwhelming and dominate the output. The cutoff to overcome length bias for calculation of CAI and ASI was determined at 60 nt (see S2 Fig).

### Tandem amplification of genes

Tandem gene arrays were identified employing the method as described by Despons et al. [35]. All-against-all search of CDSs from each chromosome was performed using BLASTn [36] algorithm. Conditions to count alignments as tandem copies were as follows–the percentage of identical matches in local alignment of two annotated genes had to exceed 90%, alignments had to reciprocally cover at least 70% of each other’s CDS length. Lastly, the spacer sequence separating two suspected tandem gene copies contained less than ten spacer genes. For a detailed discussion of our analyses of tandem genes in *T*. *congolense* see S1 Text.

### Analyses and plots

All linear correlations and plots were done using R v3.2.3 standard package. Each correlation contains the Pearson or Spearman coefficient *r* and the number of observational units *n*. If not otherwise specified, scripting for parsing the data and the results of analyses was done in Python, Java, or AWK. Gene ontology (GO) term analyses were carried out using GOSeq [37], which generates a list of enriched and depleted GO terms in a given set of genes with a respective significance level (p-value).

## Results and discussion

### Characterization of uORFs

In this work, a total of 31,149 uORFs were annotated computationally for the first time in the genome of *T*. *congolense* (see S1 Data). They were identified in 5’ UTRs of 2470 genes, which comprises 29.6% of all protein-coding genes with annotated 5’ UTRs in the parasite’s genome (see Materials and Methods). Considering only the longest uORF variant as the representative sequence for each stop codon (see Materials and Methods), it leaves 15,348, 13,467, 15,920 and 15,920 non-redundant uORFs identified in BSF, PCF, EMF and MCF, respectively (18,511 unique uORFs in total). Assumedly, the ribosome assembles at the first start codon it encounters and hence whenever uORF translation takes place, the longest possible uORF variant is translated [17]. Alternative trans-splicing in trypanosomes can lead to 5’ truncations and thus can cut uORF start or stop codons from pre-mRNA. Depending on the position of stage-specific trans-splicing sites (see Materials and Methods), this can cause different uORF variants (all sharing the same stop codon) to be the longest possible uORF in certain stages. However, only 68 uORFs (see S1 Data) show different longest variants throughout the life cycle. In the more common case that all start codons or even the uORF's stop codon are cut out, the respective sequence is not present at all in the observed transcript (see S1 Fig).

### uORF features

The annotated uORFs (non-redundant set) comprise a median length of 51.0 nt (Q1 = 24 nt, Q3 = 105 nt) and are situated at a median distance of 447.0 nt upstream of a respective CDS start codon (Q1 = 183.5 nt, Q3 = 782.0 nt). Codon usage in *T*. *congolense* uORFs is not randomly distributed, but shows an extensive bias especially pronounced in the most frequently used amino acids. The top five most frequently used amino acids, namely leucine, serine, arginine, valine and alanine, make up 39.1% of all uORFs, and are furthermore established among the top eight amino acids with most distinct codon bias (S3 Fig).

Due to a considerable bias by sequence length, calculation of CAI (Codon Adaptation Index) and ASI (Amino Acid Similarity Index, see Materials and Methods) are only relevant for the subset of uORFs longer than 60 nt. Throughout the subset of shorter sequences, the influence of each individual codon and amino acid is overwhelming when calculating a uORF’s mean composition–for short uORFs < 60 nt the CAI and ASI correlate with sequence length (r = 0.88, r = 0.8, respectively, see S2 Fig). The complementary subset of longer uORFs achieves relatively low CAI (median = 0.21, Q1 = 0.20, Q3 = 0.23) and high ASI (median = 0.0281, Q1 = 0.0187, Q3 = 0.0403) values without length bias (r = 0.34, r = 0.33 correlation with sequence length, respectively), as an expression of the higher variety in codon and amino acid usage of uORFs, when compared to CDSs.

**Fig 1:**
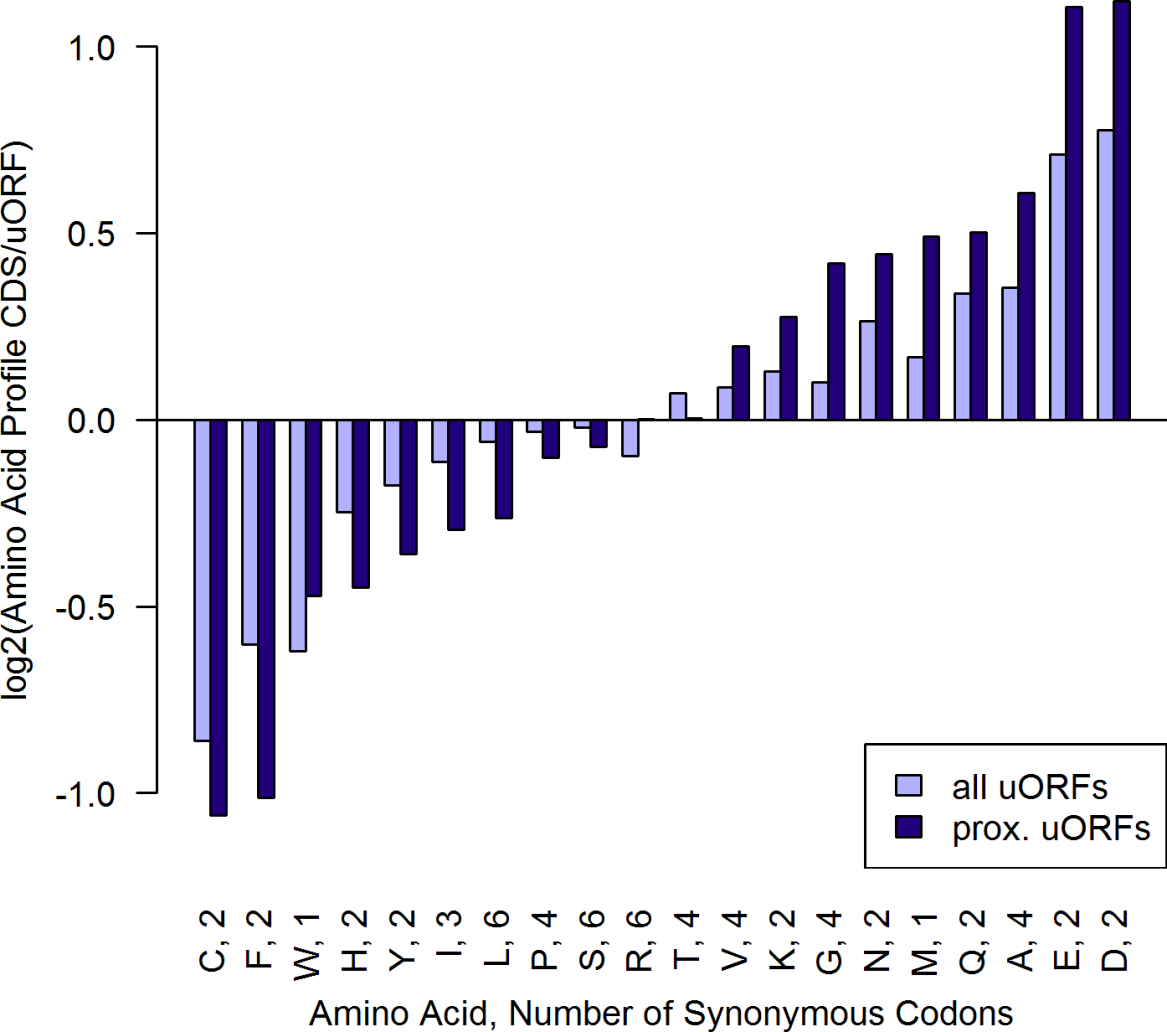
Comparison of amino acid usage among CDSs and uORFs. Y-axis values were calculated as the log2 of the ratio of amino acid usage in CDSs and uORFs for each individual amino acid, taking BSF stage as an example (for all stages see S9 Fig). A positive value represents amino acids expressed more frequently in CDSs than in uORFs and vice versa. The stop codon "s" is expressed by a factor 17.5 more frequent in uORFs (due to length bias). Length bias for methionine (start codon) is eliminated by not counting the first ATG of each sequence. To keep a y-range allowing comparison of different amino acids, "s" wasn’t included into the plot. The amino acid usage bias is especially pronounced in the subset of proximal uORFs (uORF to CDS distance < 80 nt). (n_alluORFsBSF_ = 15,348 n_prox.uORFsBSF_ = 1,385).

**Fig 2:**
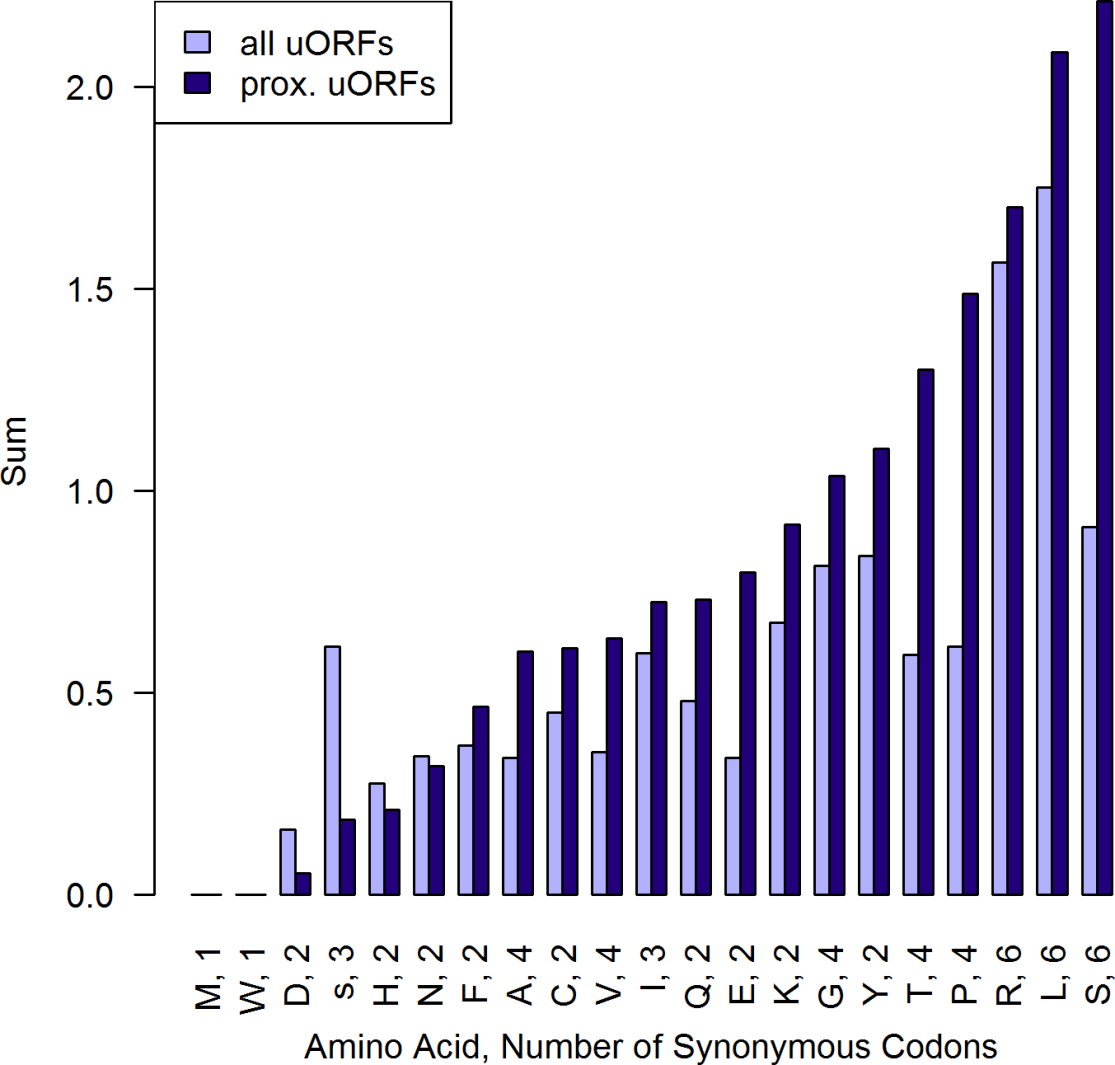
Sum of absolute log2(codon frequency CDS/uORF). For each subgroup of synonymous codons, the sum of the absolute value log2 of codon usage ratio CDS/uORF was calculated, taking BSF stage as an example (for all stages see S10 Fig). Greater values represent subgroups of synonymous codons that are relatively more biased in their usage between uORFs and CDSs. Particularly proximal uORFs show distinct variation from the CDSs codon usage. (n_alluORFsBSF_ = 15,348 n_prox.uORFsBSF_ = 1,385).

To verify if the measured uORF properties do not actually depict general features of 5’ UTR, significant distinctions between uORF and non-uORF parts of 5’ UTR were identified: amino acid and codon composition of non-uORF 5' UTR were calculated in all three frames and compared to uORFs (see S4 Fig). It appears that uORF sequences differ significantly from 5’ UTR parts, which are not framed by a start and stop codon (= non-uORF 5’ UTR). Codon and amino acid bias between uORF and non-uORF sequence in 5’ UTR presents a similar extent as the respective bias between uORF and CDS sequence (see Fig 1 and Fig 2).

### CDS features and uORF density

CDSs in the set of 8,345 *T*. *congolense* genes with annotated 5’ UTRs (see Materials and Methods) present a median length of 1,137.0 nt (Q1 = 714.0 nt, Q3 = 1,758.0 nt) and their respective 5’ UTRs have a median length of 127.0 nt (Q1 = 50.0nt, Q3 = 368.0 nt) (see S5 Fig). 2,470 genes contain at least one uORF, while the median value among the uORF-containing genes is 6.0 uORFs per gene (Q1 = 1.0, Q3 = 11.0) (see S5 Fig). A major fraction of the uORF-containing genes harbors only a single uORF (28.5%). Evaluating changes in uORF quantity throughout the life cycle, the EMF stage presents the largest proportion of uORF-containing genes: 90.7% of genes, which possibly enclose a uORF in any of the life cycle stages (2,470), present a uORF in EMF. 5’ UTR length correlates with the number of uORFs (r = 0.88), meaning larger transcript leaders generally accommodate a greater number of uORFs (see S5 Fig). Median uORF density in uORF containing 5’ UTR, considering the maximum transcript leader length and maximum number of unique uORFs throughout the life cycle, accounts to 1.1 uORFs per 100 nt (Q1 = 0.8, Q2 = 1.4).

Codon and amino acid usage are extremely biased in *T*. *congolense* CDSs. The top five most frequently used amino acids leucine, alanine, serine, valine and arginine represent 40.3% of the CDS sequence, along with leucine, valine and arginine being the three most biased subgroups of synonymous codons (see S5 Fig). The biased usage of an optimized set of codons and amino acids is indicated by the calculation of relatively high CAI (median = 0.80, Q1 = 0.79, Q3 = 0.82) and low ASI values (median = 0.0050, Q1 = 0.0030, Q3 = 0.0083). For a detailed discussion of CDS properties see the S5 Fig.

### ORF length

CDSs and uORFs obviously differ significantly in their sequence length (CDS median length 1137.0 nt, Q1 = 714 nt, Q3 = 1758 nt, uORF median length 51.0 nt, Q1 = 24 nt, Q3 = 105 nt, see S3 Fig and S5 Fig). The CDS length is determined by the respective peptide’s length and thus cellular function. The length of a uORF and more specifically the duration of elongation phase is considered to be an influential feature on its repressive potential [17,31].

### Amino acid usage

**Fig 3:**
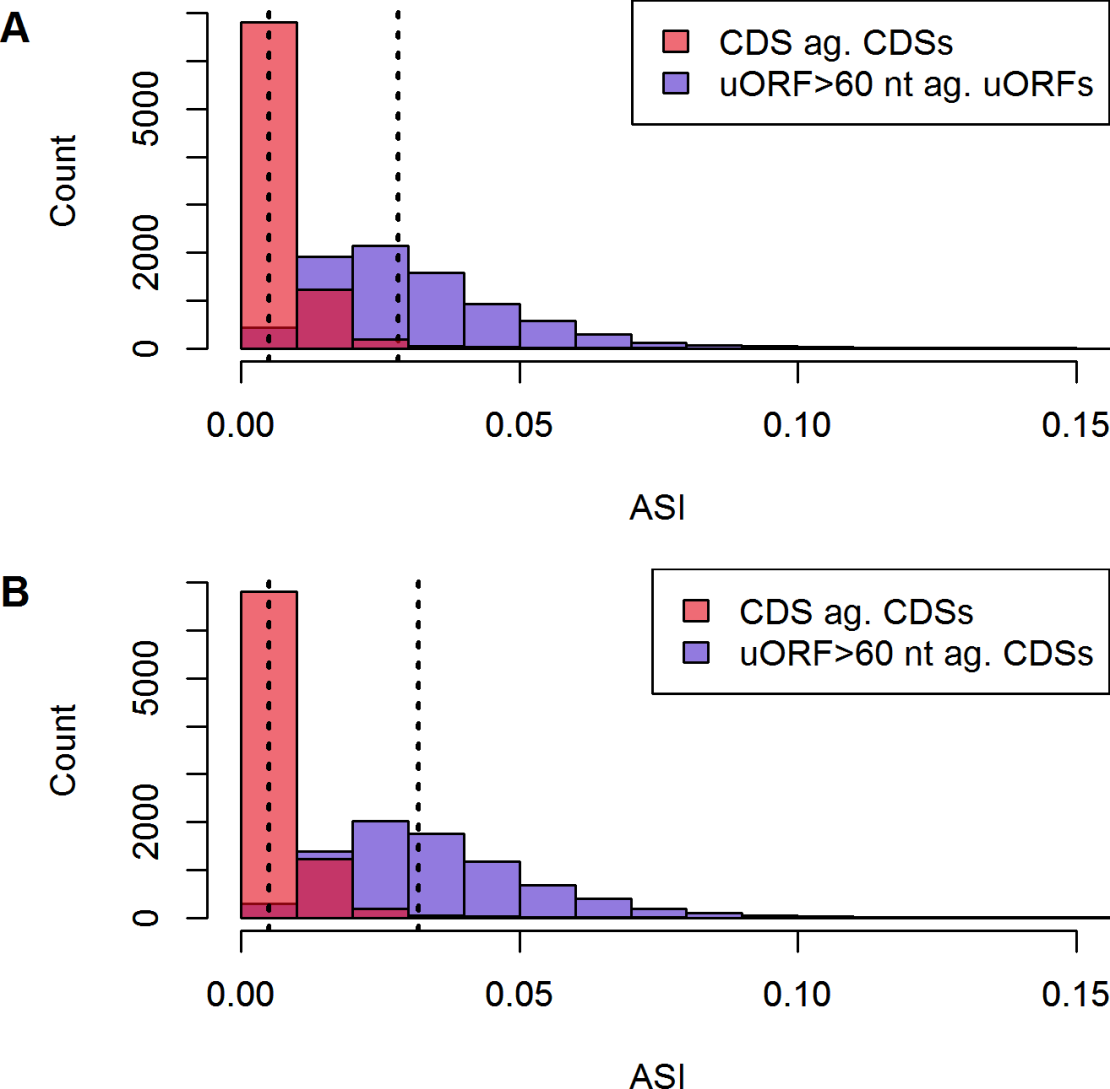
ASI CDS against CDSs and uORF>60 nt ag. uORFs and CDSs. Due to the extensive length bias, ASIs are only calculated for uORFs longer than 60 nt. CDSs and uORFs present significantly different distributions of ASI (non-paired Wilcoxon test, p<0.05, p<0.05). Median (dashed lines) ASI for uORFs against uORF background and CDSs is 0.0281 and 0.0050, respectively (**A** plot). uORF ASI against CDSs achieves a higher median than calculation against the intrinsic subset (0.0319, **B** plot). Additionally, uORF distribution comprises a broader range of ASIs than CDSs. CDSs employ a distinct set of amino acids, while uORFs display larger variability, still including some sequences with highly optimized ASIs. Calculation of uORF ASI against CDSs reveals the disparity of both subsets. (n_CDS_ = 8,345, n_uORF_ = 8,156).

To measure the differences of amino acid usage between CDSs and uORFs, the log2 of the ratio of each amino acid's frequency in CDSs divided by its respective frequency in uORFs was calculated and depicted in Fig 1. Start and stop codon frequencies are biased by sequence length. This length bias was dismissed for methionine (start codon) by excluding the first “M” in every sequence from the analysis, while the stop codons were not considered for amino acid profile comparison. There is a different amino acid usage in *T*. *congolense* CDSs and uORFs (see Fig 1), which is especially pronounced comparing CDSs and proximal uORFs. In particular the amino acids aspartic acid, glutamic acid, cysteine and phenylalanine are biased (2.2 and 2.2 fold in CDSs compared to proximal uORFs, 2.1 fold and 2.0 fold in proximal uORFs compared to CDSs, respectively). To determine, if a distinct, optimized amino acid profile can be extracted from CDSs and uORFs, the ASI was calculated (see Fig 3). As illustrated in Fig 1 and Fig 3, the usage of amino acids is non-randomly distributed in *T*. *congolense* uORFs as well as CDSs. In genuine peptide-coding CDSs with annotated 5’ UTR, this amino acid usage bias can be explained by their biological function only allowing a certain set of amino acids. On the contrary, alternate usage of functionally similar amino acids permits some variation in amino acid profiles, especially in non-critical regions for peptide functionality [38]. In this limited range, a tendency towards usage of metabolically less costly amino acids has been discussed [38]. Regarding hypothetically truly synonymous codons, evolutionary pressure for selection bias does not arise from the function that the peptide product serves, which does not change when replacing an amino acid codon with another synonymous one, but from the costs of its components [39]. Varying metabolic constraints are set up by highly diverse direct costs of amino acid biosynthesis, such as NADH or ATP consumption [38].

**Fig 4:**
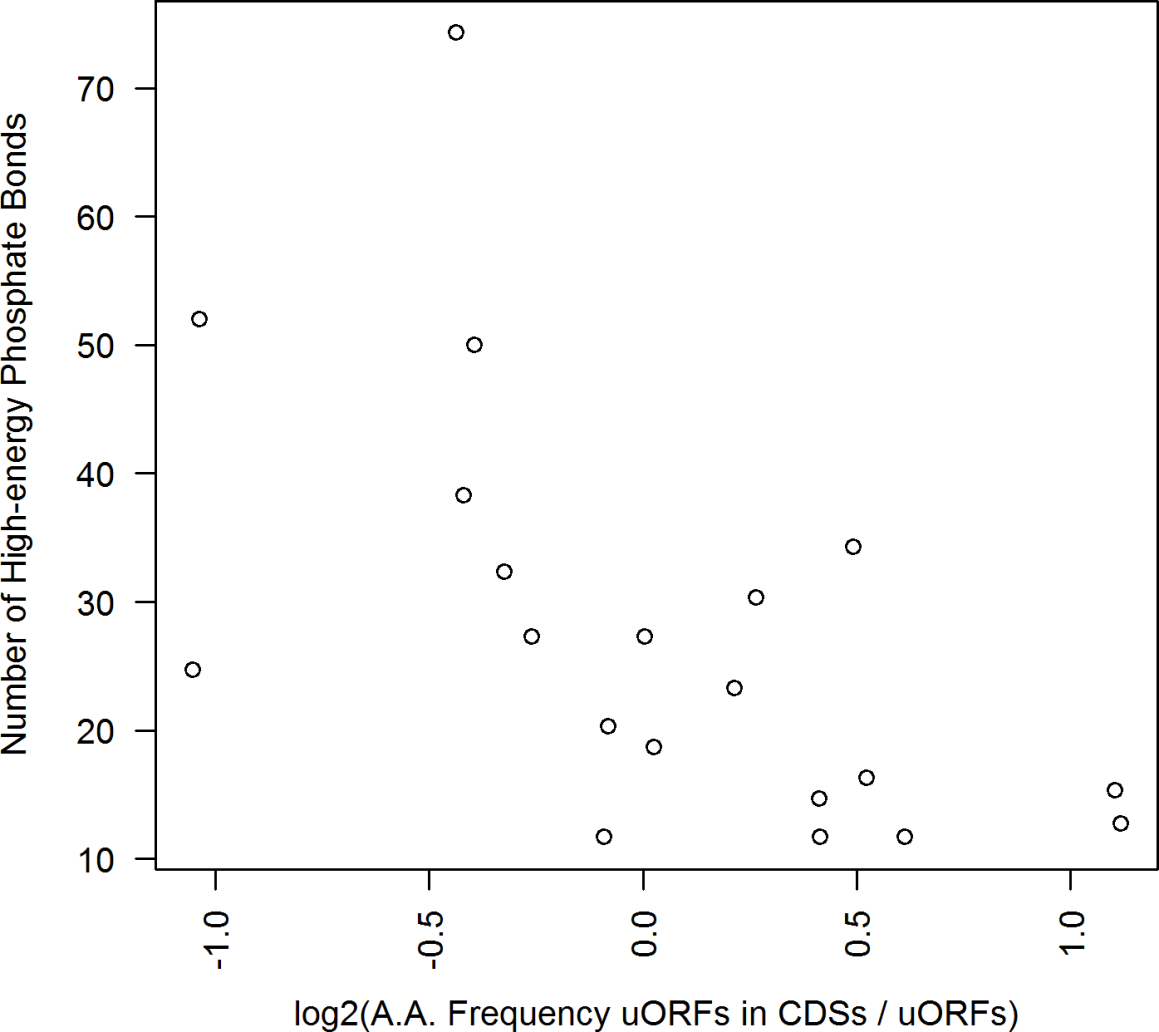
Amino acids with high energetic constraints are overrepresented in proximal uORFs. **Each of the 20 proteinogenic amino acids is shown by a scatter plot. Positive x-values represent amino acids, which are contained more often in CDSs compared to proximal uORFs and vice versa. Generally,** metabolically costly amino acids are used more frequently throughout proximal uORFs. (n_prox.uORFs_ = 1,628).

It has been shown that conserved uORFs encounter weaker evolutionary selection of amino acid usage than other genic sequences [40]. The data presented here agrees with these findings, demonstrating higher ASI values and thus greater variation of amino acid profiles in uORFs as compared to CDSs (see Fig 3). Assuming a random base sequence in uORFs, the amino acids with most synonymous codons such as leucine, arginine and serine (six synonymous codons each) would be preferred in uORFs compared to CDSs. In fact, they are overweighted in uORFs, but only to a small extent (see Fig 1). Surprisingly, the most frequent amino acids in uORFs are cysteine (C) and phenylalanine (F) with no more than two synonymous codons. This observation is particularly pronounced in proximal uORFs (see Fig 1). Furthermore, these two most biased amino acids are among the rarest in CDSs (see S5 Fig) and individually used on average with a frequency of merely 2.9% in protein-coding CDSs. These features make sense in the light of the repressive nature of uORFs: assuming that the biosynthesis of peptides is one of the major duties for living cells, the metabolism of *T*. *congolense* should aim at optimum translation of its CDSs [41]. Our data suggest, that uORFs are under an evolutionary pressure to express these distinctive amino acids, which are not efficient to serve the peptide metabolism of *T*. *congolense* and thus are underrepresented in CDSs. Fig 4 illustrates that biosynthetically highly energy consuming amino acids [38] are less frequent within CDSs and relatively overrepresented in uORFs.

The organism provides the respective costly tRNAs at lower abundance [38], preventing inefficient use of resources. Ultimately a lower level of tRNA corresponds to a lower elongation speed of the peptide chain [41]. Summing up, the preferred usage of exclusive, costly amino acids in uORFs might cause a slower, inefficient translation and in the end contribute to the repressive potential of the respective sequences. This hypothesis might be particularly relevant for uORFs inside the critical window of 80 nt upstream from CDS start [24,25].

### Codon usage

**Fig 5:**
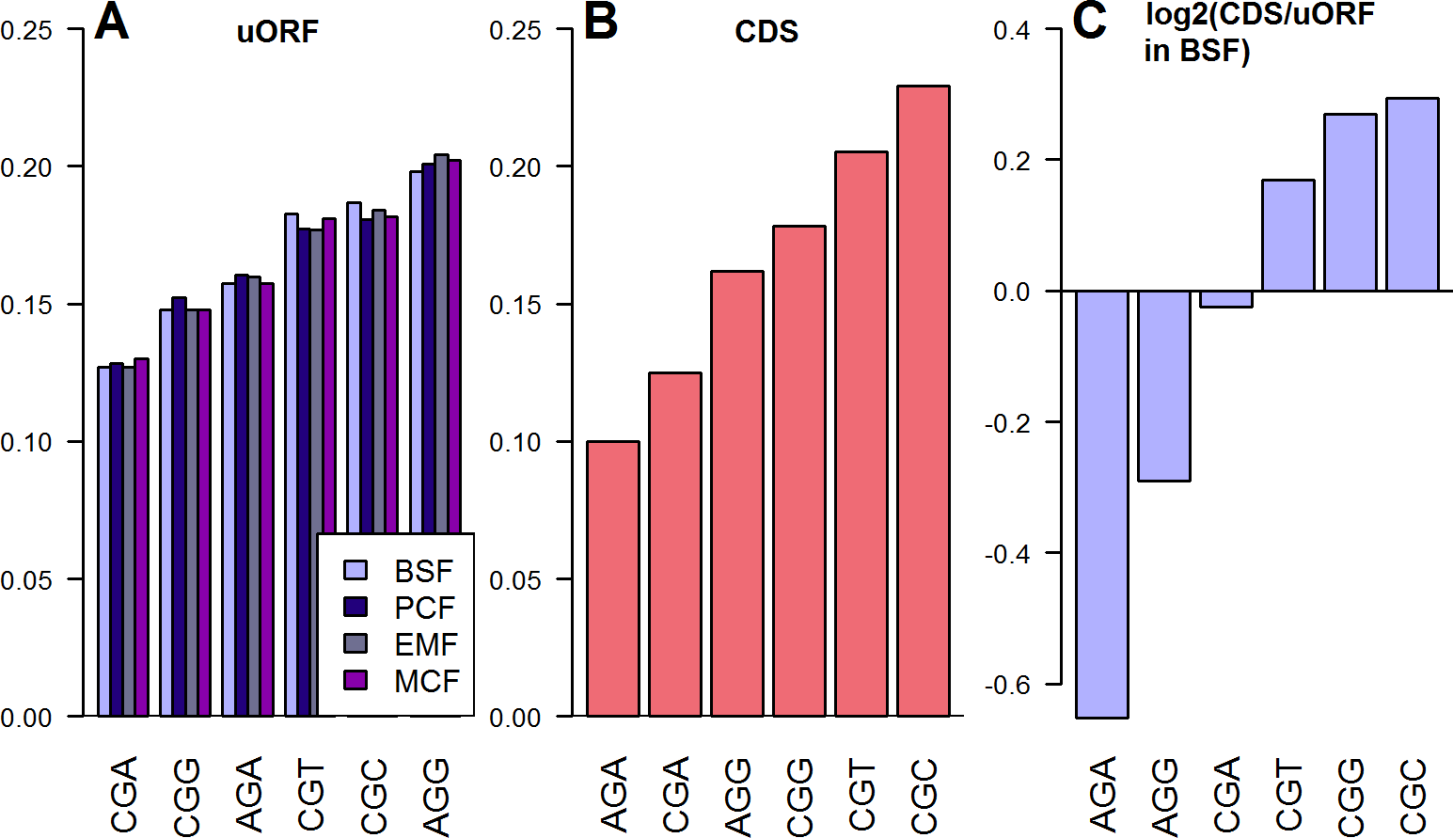
Codon usage bias in uORFs and CDSs on the example of arginine. For each amino acid, a subgroup of synonymous codons was composed (CGA, AGA, CGG, CGT, CGC and AGG for arginine). Subsequently, the normalized usage frequency of each codon was calculated. (**A**) Among the uORFs in all the four life cycle stages of *T*. *congolense*, AGG is the most frequently used codon for coding arginine and represents 20.0% of arginines. (**B**) Among the CDSs, the respective “preferred” codon is CGC, which accounts for 22.9% of arginines. (**C**) For a comparison of the normalized codon usage in CDSs and uORFs, BSF stage was used as an example. i.e. for coding arginine, CGC usage is 1.22 fold higher in CDSs, while AGA is employed 1.49 fold higher in uORFs.

To compare the codon usage in uORFs and CDSs, the log2 of the ratio of each codons normalized usage frequency in CDSs divided by the usage in uORFs was calculated (see Fig 5 as an example). Normalized codon usage is significantly biased between CDSs and uORFs for 59 out of 64 codons (chi-squared test, p<0.05, see S7 Fig). In order to measure the overall magnitude of codon usage bias between uORFs and CDSs, the sum of the absolute values *log2 (codon frequency CDS/codon frequency uORF)* was calculated for each subgroup of synonymous codons (see Fig 2 and Fig 5 for explanation). The subgroups of synonymous codons for serine, leucine and arginine are overall most biased in their usage between uORFs and CDSs. Methionine and tryptophan are only represented by a single codon and therefore do not show any difference in codon usage (y-value = 0). For coding of amino acids present on the left end of Fig 2, uORFs and CDSs employ a similar set of synonymous codons. Generally, codon usage bias is more pronounced between CDSs and proximal uORFs.

**Fig 6:**
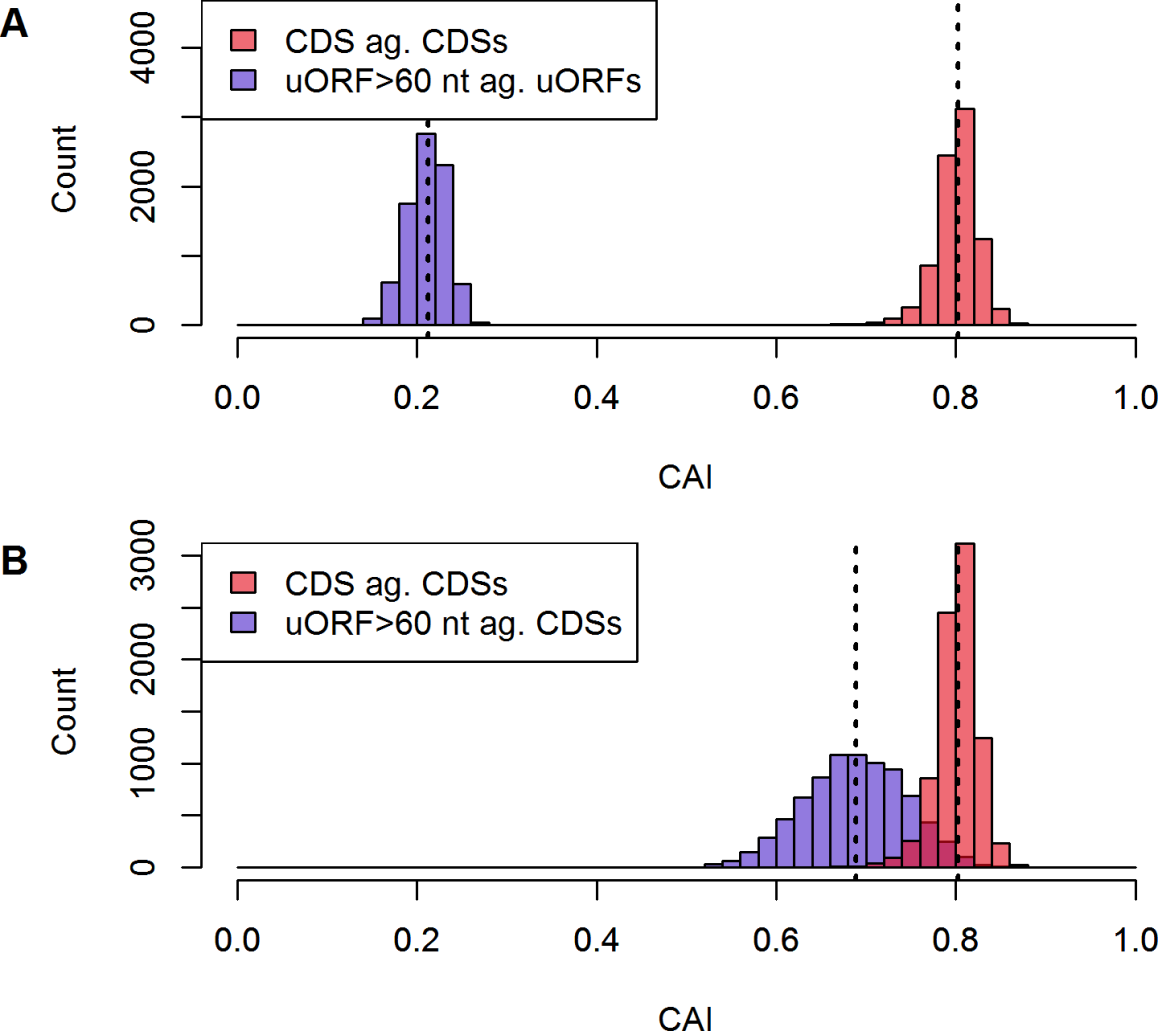
Distribution of CAI throughout uORFs and CDSs. (**A**) uORFs against uORF background achieve significantly lower CAIs compared to CDSs. Dashed lines represent the median CAI values for both sets (0.21 and 0.80 for uORFs and CDSs, respectively). There is not a single sequence overlapping the other subset’s CAI values, when calculated against the inherent subset. (**B**) Calculating the CAI for uORFs against CDS background results in an overlap of both subset’s CAI range. The difference of CAIs in both sets remains significant (median CAI 0.69 and 0.80, non-paired Wilcoxon test, p<0.05). (n_CDS_ = 8,345, n_uORF_ = 8,156).

To estimate preference of optimum codons throughout the sequences, the CAI was calculated for uORFs and CDSs (see Fig 6). CDS and uORF CAIs differ significantly (median CAI 0.80 and 0.21, respectively). Throughout uORFs, the employed algorithm for CAI calculation does not determine a distinct set of preferably used codons and thus outputs low CAIs for all uORFs. Remarkably, not a single CDS or uORF CAI overlaps the respective other subgroup, when calculated against the inherent subset. Subsequently, the CAI was determined for uORFs against CDS background, which represents the uORFs resemblance of the species-specific metabolically optimal set of codons (see Fig 6). Calculating CAI values of uORFs against CDSs results in an overlap of CAIs from both subgroups. The biological function of these overlapping uORF sequences with relatively high CAI can be interpreted in two ways: either, judging from their set of efficient codons, the above-mentioned sequences were evolutionary selected to mediate a low translation repressive potential, or they correspond to wrongly annotated CDSs and hence resemble the characteristic CDS codon usage bias.

### Highly expressed genes are released from uORF repression

In order to analyze uORFs of highly expressed genes, three approaches to defining such subsets were followed. The first subset includes tandem amplified genes. In *T*. *brucei*, tandem amplification has been suggested to augment the expression at the level of individual genes, which is otherwise not possible due to absence of single gene promoters [42]. In general, genes augmented in tandem arrays are considered be among the most highly expressed in trypanosomes [11]. Throughout the subset of genes with annotated 5' UTR, a total of 609 tandem amplified genes were computationally annotated (317 TGAs after excluding the *T*. *congolense* BIN pseudochromosome, which is composed of randomly ordered genomic contigs; see S1 Text). Second group of extensively expressed genes contains the CDSs scoring top ten percent of CAI values. GO term analysis, which is obligatory to confirm the biological significance of CAI calculation [34], reveals significant enrichment of the top CAI subset (p<0.05) with essential biological functions, such as translation and basic energy metabolism. A full list of enriched GO terms in the top 100 CAI genes is provided in S2 Table. The third subset comprises all genes with the proteomic data available and was chosen based on the technical details of mass spectrometry analysis (MS), where highly abundant proteins are most likely to generate a significant spectrometry signal [43].

A summary of uORFs included in the above outlined subsets of highly expressed genes is provided in Table 1. Typically, uORFs are regarded to potentially repress translation of the downstream CDS, if they are proximal to or overlapping the CDS start codon. On the contrary, the subsets of important genes contain significantly lower numbers of high impact uORFs, ultimately preserving their expression level (see Table 1). The proximal uORFs still contained in 5’ UTRs of important genes are on average situated further upstream, providing the ribosome with a longer gap after their translation to regenerate initiation factors, eventually mediating less repression of translation (see Table 1). CDS overlapping uORFs, which potentially shunt the ribosome over the CDS start codon, are very rare in highly expressed genes (0.06 to 0.09 overlapping uORFs per gene, see Table 1). After all, largely expressed genes contain longer uORFs than the complementary subsets. This counter-intuitive correlation cannot be explained by the mechanical model of ribosomes gradually shedding crucial factors for reinitiation during translation, assigning a higher translation repressive potential to longer uORFs. However, recent research on uORF features has questioned the commonly accepted impact of uORF sequence lengths on its repressive capability [21,29]. Thus the numbers in Table 1 are in accordance with this contemporary view.

**Table 1:** Summary of uORFs preceding highly expressed genes.

	tandem amplified genes (no BIN) (n = 317 CDSs)		available proteomic data (n = 1,693 CDSs)		top 10% CAI (n = 835 CDSs)	
	tandem amplified	ratio to comp. set	prot. av.	ratio to comp. set	top CAI	ratio to comp. set
mean number of **proximal** uORFs per CDS	0.14	1.39	0.09	2.64	0.11	1.88
mean number of **overlapping** uORFs per CDS	0.09	1.47	0.06	2.32	0.07	2.02
**proximal** uORFs’ median **length**	75.0 nt	0.72	67.5 nt	0.77	87.0 nt	0.58
proximal uORFs’ median **uORF to CDS distance**	37.0 nt	1.05*	42.0 nt	0.93	42.0 nt	0.93*
mean number of proximal **in-frame Ms**	0.13	1.74	0.09	2.95	0.09	2.69
median in-frame proximal **M to CDS start** distance	45.0 nt	0.89*	47.0 nt	0.85*	52.0 nt	0.77
proximal long uORFs’ median **CAI** against CDS	0.73	0.94	0.69	1.00*	0.69	0.98
non-proximal long uORFs’ median **CAI** against CDS	0.70	0.99	0.69	1.00*	0.70	0.98
proximal long uORFs’ median **ASI** against CDS	0.0314	1.12*	0.0414	0.84	0.0381	0.96*
non-proximal long uORFs’ median **ASI** against CDS	0.0334	0.97*	0.0328	0.98*	0.0333	0.97*

Subsets of highly expressed genes were defined by their tandem amplification (excluding BIN chromosome), availability of proteomic data and top CAIs. In general, extensively expressed genes contain less overlapping and proximal uORFs (uORF to CDS distance < 80 nt), which are longer and further upstream of the CDS start codon. Furthermore they avoid the translation of methionine close (< 80 nt) to the CDS start codon. CAI and ASI ratios for proximal, long (> 60 nt to overcome the length bias) uORFs are mostly not significant. Factors marked with * are not significant (non-paired Wilcoxon test, p<0.05)

### uORF codon and amino acid profiles might affect gene regulation

**Fig 7:**
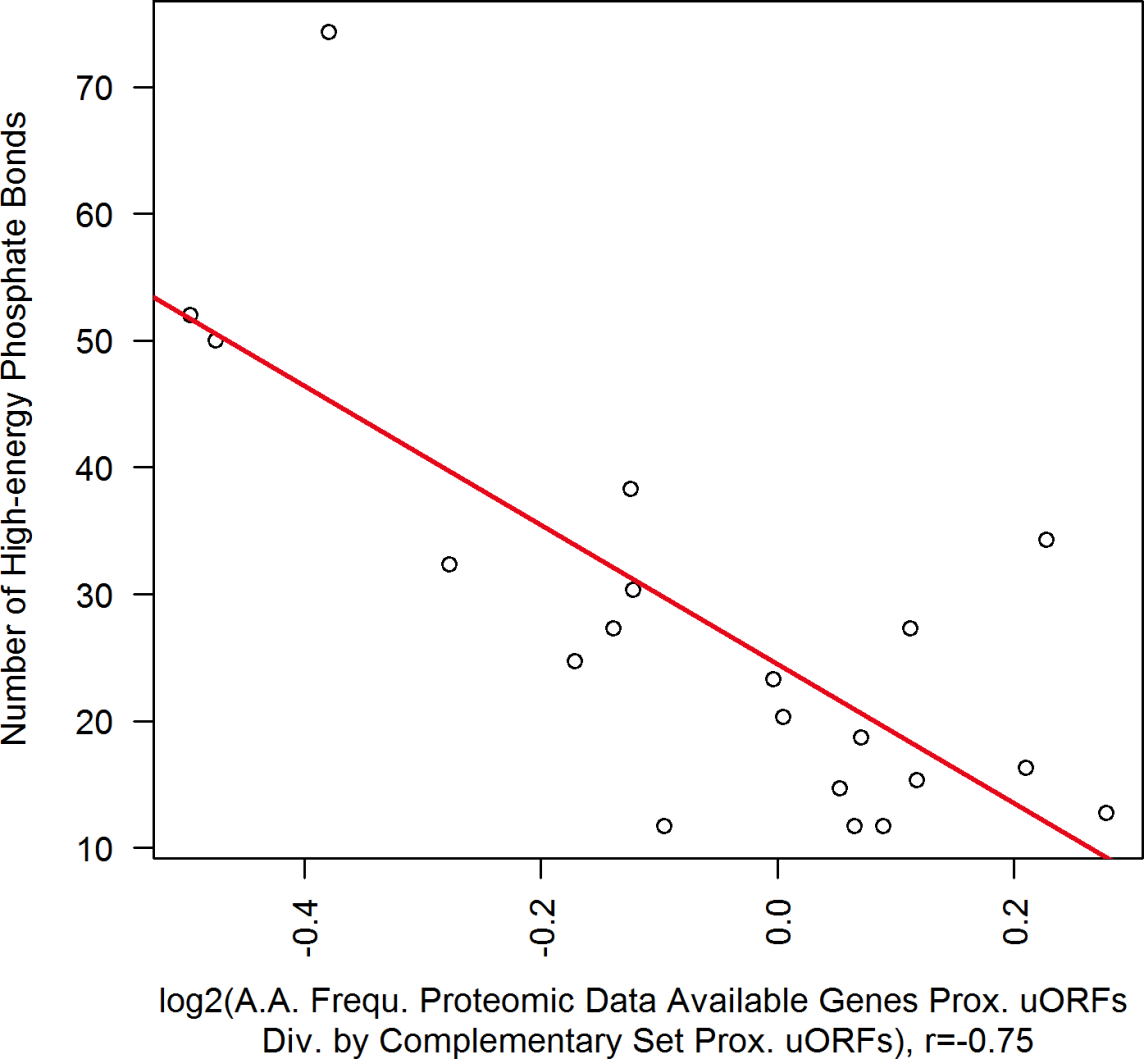
Proximal uORFs in highly important genes contain optimized amino acids. Each of the 20 proteinogenic amino acids is represented by one scatter. The subset of genes, which provided significant signal for acquisition of proteomic data, are presumably among the most translated (see text for details). The x-axis plots are the quotient of amino acid frequencies throughout uORFs in the subset of genes with available proteomic data. Positive values represent amino acids more frequently used throughout uORFs in the extensively translated genes, and mostly correspond to metabolically low-cost amino acids (bottom right quadrant of the figure). The figure employs the set of genes with available proteomic data exemplary as an extensively translated subset. Respective *r* values for tandem and top CAI genes, which are also presumably among the most translated genes, are -0.75 and -0.72. (Of 1628 proximal uORFs, n_tandem_ = 45, n_proteomic_ = 159 and n_topCAI_ = 91 uORFs are included in the three subsets of highly expressed genes).

Codon and amino acid usage seem to lack enough attention as one of the important features defining the uORF’s repressive potential in the recent publications [25,26,44]. On the contrary, it is commonly accepted that CDSs using a set of optimized codons [45] and amino acids [46,47] are translated more efficiently and accurately, because they take advantage of the species-specific developed well-established metabolic track and for instance correspond to the most abundant tRNAs. In case of uORFs, primarily the factor of translation efficiency is crucial, considering every stalling of ribosomes on 5’ UTR to diminish chances of reinitiation [17] and shift the equilibrium between CDS translation and mRNA decay in favor of the latter [4]. Based on our observations, we suspect that the usage of common, low-cost amino acids, might facilitate uORF translation and ultimately preserve translation of highly expressed genes. Throughout the *T*. *congolense* genome, amino acid profiles of proximal uORFs in highly translated genes relatively resemble metabolically economic components (see Fig 7). However, ASI calculation of uORFs against CDSs is mostly not significant. Regarding codon usage, CAI against CDS background ratios differ significantly in tandem and top CAI genes against the respective complementary subsets, regardless of the respective uORFs position in 5’ UTR (see Table 1). Lower CAIs for uORFs in extensively translated genes indicate the bias of codon usage towards an optimized CDS profile throughout the respective uORFs. CAIs against CDS background also show a significant bias throughout non-proximal uORFs (also see S8 Fig), which fall outside of the postulated 80 nt translation repressive window [17]. It is known that replacing optimum codons by synonymous rare codons in a CDS remarkably decreases mRNA stability [28]. This concept could be transferred to uORFs [29], even those which are not situated in the proximal window of 80 nt uORF to CDS distance, enabling them to additionally influence the above-mentioned equilibrium in favor of mRNA decay and repressing protein expression from uORF containing mRNAs.

### Highly expressed genes avoid translation of methionine shortly upstream of the CDS

Most remarkably, in-frame methionine is extensively depleted proximal to highly translated CDS’s start (up to 2.95 fold, see Table 1). On top, the in-frame M’s are on average situated further upstream from the CDS start codon. This might be a uORF sequence equivalent to maintain the stock of Met-tRNA, in particular shortly before the crucial moment of reinitiation. Depletion of AUGs in 5’ UTR region close to the CDS start in all three frames has already been noted [21,48].

### Translation efficiency is stage specific

**Fig 8:**
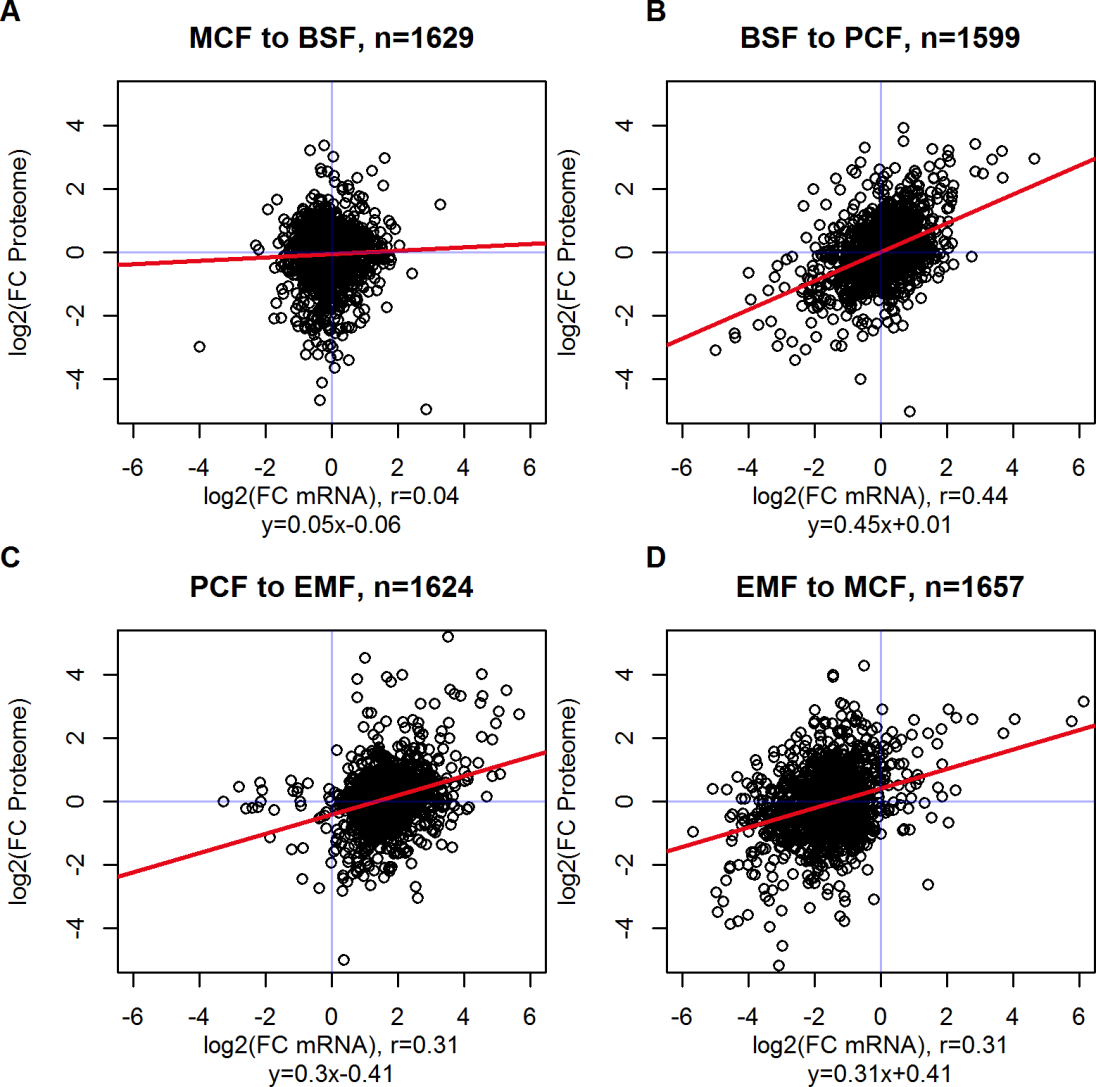
Correlation of mRNA and protein abundance. The correlation between transcript level and protein output is low, opening up a corridor for post-transcriptional gene expression regulation. The figure depicts comparison of relative fold changes (FC) of expression values between the stages. Translation efficiency is represented by the slope of the regression line. (**C**, **D**) From PCF to EMF and EMF to MCF the scatters are shifted on the x-axis, indicating an accumulation of transcripts in EMF, possibly due to an increased mRNA half-life.

Gene expression regulation in trypanosomes is realized predominantly post-transcriptionally. However, in Fig 8 a weak positive correlation between mRNA and proteome abundance in all four stage changes can be noticed, assigning a generally augmenting effect of transcript level on protein output and opening a narrow corridor for a pre-transcriptional control. Translation efficiency, as a device of post-transcriptional control, is the ratio of the amount of protein translated per mRNA unit and represented by the slope of the regression line. The Pearson correlation coefficient *r* is a measure for magnitude of potential post-transcriptional gene expression regulation. Assuming an *r* equal to 1, the protein output only depends on mRNA levels, disproving any post-transcriptional regulation. Remarkably, from PCF to EMF and consecutively from EMF to MCF translation efficiency doesn’t change (slope of 0.3 and 0.31, respectively), but the whole scatter plot is shifted on the x-axis: from PCF to EMF, mRNA change is predominately positive and vice versa, from EMF to MCF. EMFs accumulate transcripts and hence contain an absolute higher number of mRNAs than the remaining stages. The stage-specific absolute numbers of transcripts from the RNA-seq experiment are significantly higher in EMF stage and support this thesis (median in BSF = 39, PCF = 40, EMF = 120, MCF = 46, paired Wilcoxon test, p<0.05, n = 8,345 CDSs). Subsequently, the mRNAs are degraded before transition to MCF stage. Agreeing with these findings, an expanded mRNA half-life could be shown in EMFs of *T*. *cruzi* [49]. By global down regulation of RNA decay in non-dividing EMFs, housekeeping transcripts could be stabilized to ensure basic functions a life cycle stage of generally repressed metabolism [49].

### uORF position in 5' UTR defines repressive potential

**Fig 9:**
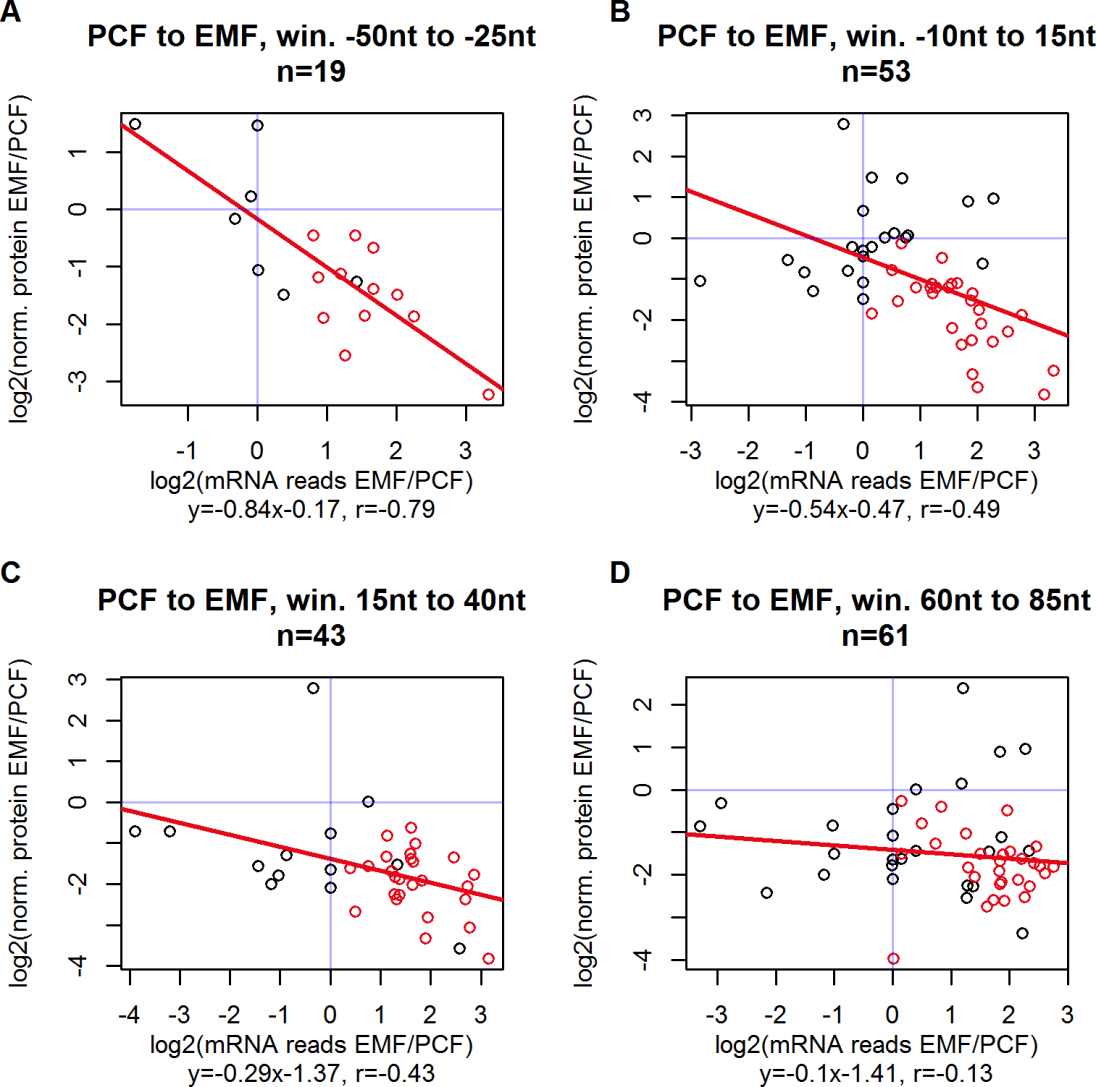
uORFs overlapping CDS mediate the highest repressive potential. During the progression of the life cycle stages, uORFs are cut out or can appear again in the transcriptome due to alternative trans-splicing and might affect translation efficiency depending on their position in 5’ UTR. The example of transition from PCF to EMF is most promising to study the repressive potential of uORFs, since EMF presents the highest absolute level of transcripts and relatively the largest amount of uORF-containing transcripts. The figure presents inter stage changes from PCF to EMF of their respective normalized protein levels (y-axis) and uORF-containing mRNA reads if the respective gene (x-axis). Panels **A**, **B**, **C** and **D** include transcripts, which contain uORFs with different uORF-to-CDS distance windows. Most scatters are located in the bottom right quadrant, meaning that during the progression from PCF to EMF uORFs are overweighted in the transcriptome and repress translation dose dependently. Red scatters indicate uORFs that are consecutively underrepresented in MCF and release translation repression again (see Fig 10). The regression lines slope can be employed as a measure, how effective overweighting a uORF correlates to protein output control. (**A**) In general, overlapping uORFs score the highest repression efficiency. (**B**, **C**, **D**) The uORF’s repressive potential decreases with increasing uORF to CDS distance.

**Fig 10:**
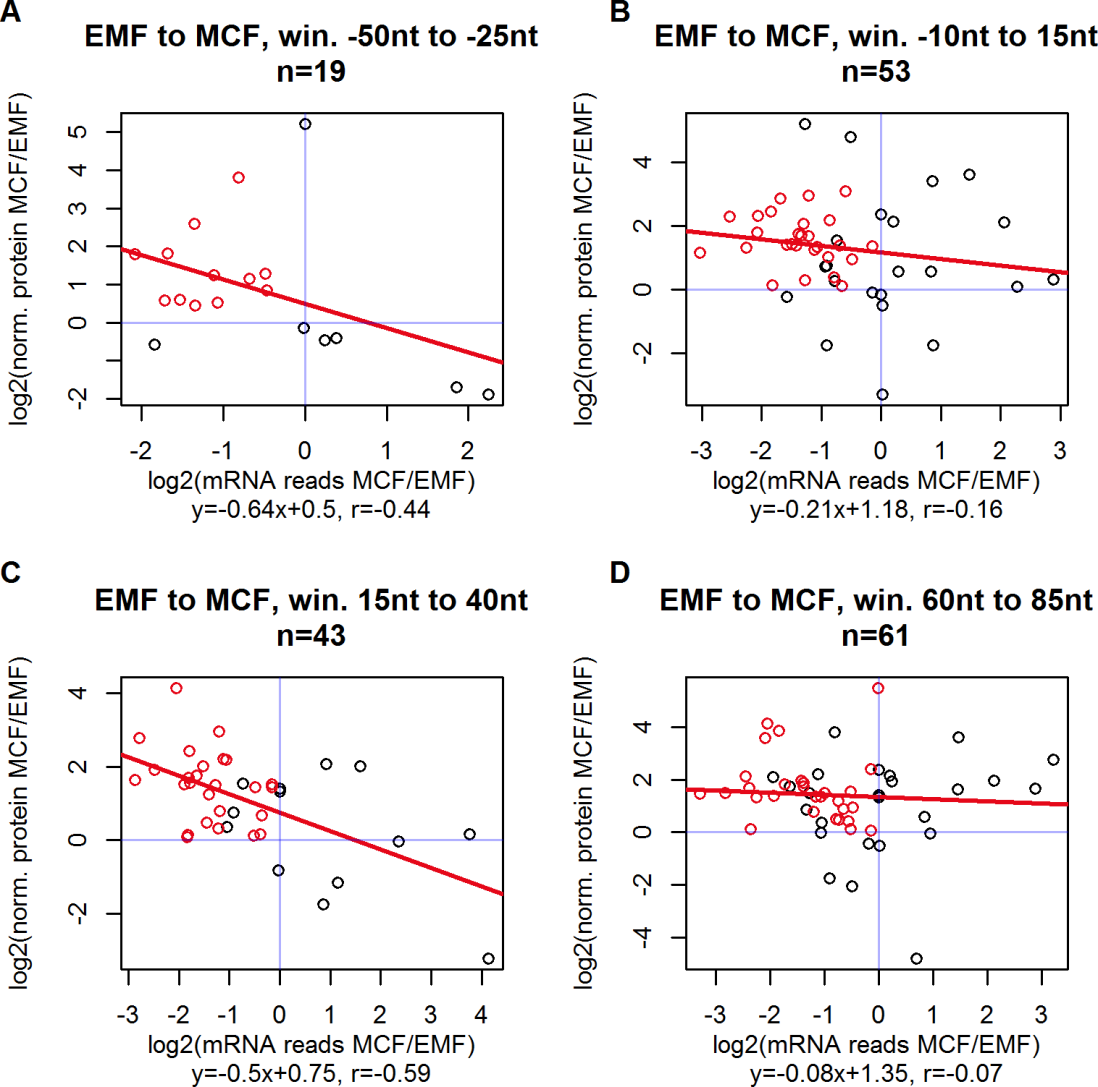
uORFs of the proteomic data subset undergo characteristic changes around EMF stage. Red scatters indicate uORFs that have been previously overweighted from PCF to EMF (see Fig 9, bottom right quadrant) and release their translation repression from EMF to MCF stage (top left quadrant). Underrepresentation of overlapping and proximal uORFs corresponds to the largest incline of protein expression (see gradually decreasing slope from panels **A** to **D**). For general graph description see Fig 9.

Assuming that uORFs can generally influence stage specific gene expression in *T*. *congolense*, some uORFs might mediate a more influential repressive effect than others, depending on their sequence specific properties. Among uORF features, the distance from uORF stop codon to CDS start codon is regarded to be among the deciding characteristics, essentially defining the repressive potential [21,26]. Fig 9 and Fig 10 explore on the example of EMF stage, how gain or loss of uORFs in the vicinity of CDS initiation sites during progression from PCF to EMF mediate a more pronounced effect on protein expression than variation of further downstream uORFs. EMF stage includes at least twice as many stage exclusively presented uORFs than all other stages (see S6 Fig), opening up a wide corridor for potential uORF-mediated translation control.

Most scatters presented in Fig 9 are placed in the bottom right quadrant, indicating that uORFs in the observed subsets are transcribed more frequently during transition to EMF and translation of the downstream CDS is consequently repressed. Especially the windows with negative uORF to CDS values, representing overlapping uORF and CDS sequences, score the most negative slopes, meaning that overweighting these uORFs results in relatively effective repression of the respective CDS. Shorter uORF to CDS distances make the event of reinitiation less likely and ribosomes translating overlapping uORFs are shunted over the CDS start codon, remaining unavailable for reinitiation [17,24]. uORF-mediated translation repression during consecutive progression from EMF to MCF stage is illustrated in Fig 10 for the same uORF to CDS distance windows. Most scatters are positioned in the top left quadrant, representing uORFs that are underweighted while completing this life cycle step and releasing their repressive potential, resulting in a more effective translation of protein.

**Fig 11:**
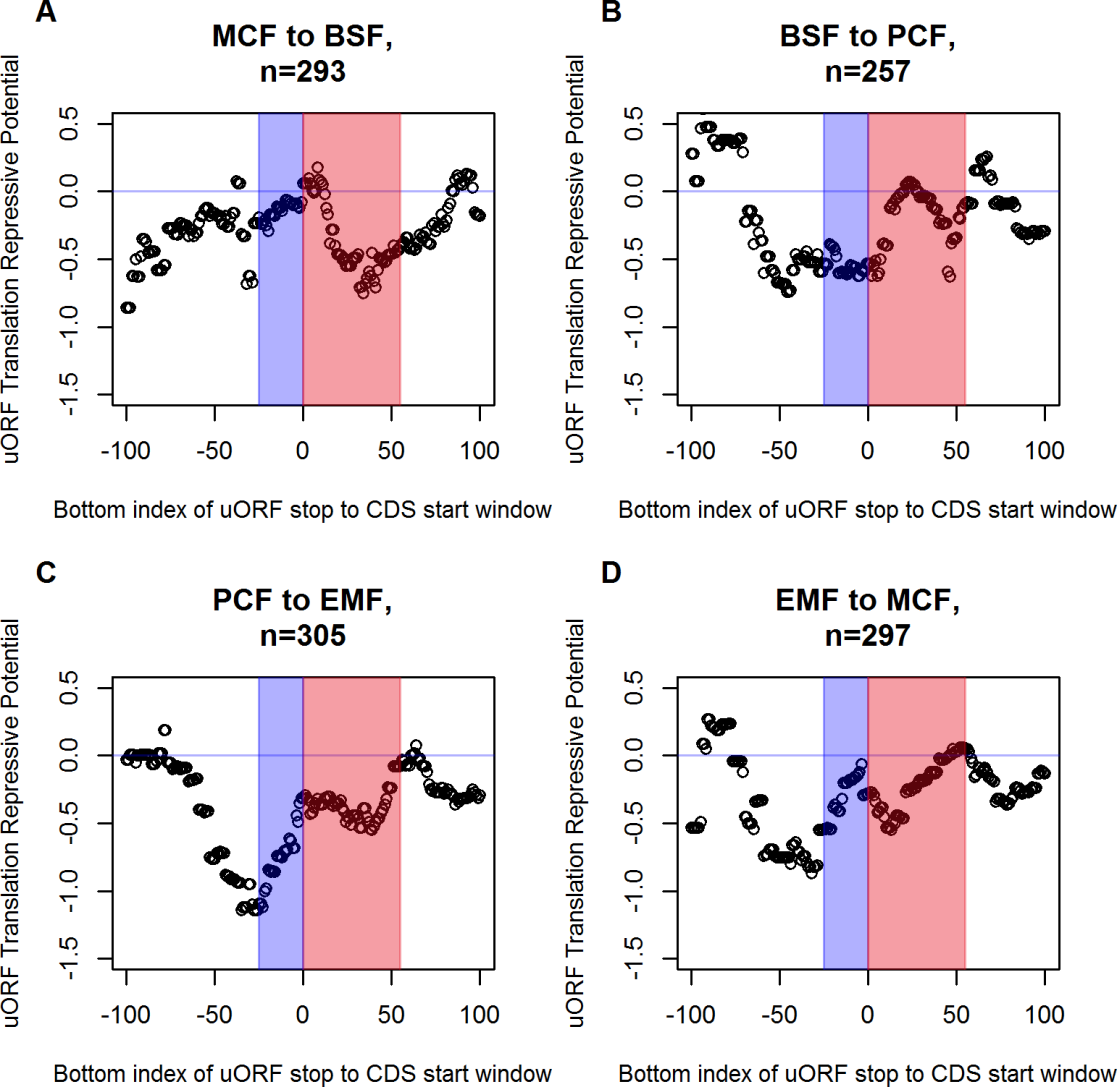
Repressive potential of uORFs depending on uORF stop to CDS start distance. **Unlike the discontinuous evaluation of uORFs-to-CDS distance effect on translation efficiency in four panels of Fig 9 and Fig 10 (25 nt wide window, bottom border at -50, -10, 15 and 60 nt), this figure represents a continuous sequence of 201, 25 nt wide uORF-to-CDS distance window, sliding from -100 nt to 100 nt uORF-to-CDS distance range (x-axis).** The y-axis represents translation repressive potential of the uORFs included in the respective uORF-to-CDS windows, calculated as the regression slope in Fig 9 and Fig 10. The blue box marks the farthest downstream window only including overlapping uORFs and plots close to maximum translation repression (min. y-value) in all four stages (see panels **A** to **D**). Further downstream windows include less *n* numbers and cause noise artifacts. The red box indicates windows including uORFs with repressive potential. Only windows containing *n*>5 uORFs are plotted to reduce noise. (**C**) Stage progression from PCF to EMF, which is promising to evaluate uORF effect on translation efficiency (see text), shows the gradual loss of translation repression when sliding the observation window upstream.

To evaluate more possible uORF stop to CDS start distances than the four windows plotted in Fig 9, and examine the other life cycle stages, 25 nt wide windows were defined starting at every uORF to CDS distance from -100 nt to 100 nt. Calculations were not done over the whole range of uORF stop to CDS start distance, because for < -100 nt, *n* numbers are getting relatively small while at higher distances, uORFs lose their repressive potential [24,25]. For each of the stage progression specific 201 resulting subsets (mean number of uORFs m_EMF_ = 35 uORFs, overall n_EMF_ = 295 uORFs), the regression line’s slope was calculated as established in Fig 9 and plotted against the position of the respective uORF stop to CDS start window (see Fig 11). An example window from -25 nt to 0 nt is marked by a blue box, including overlapping uORFs with stop codon position 25 nt downstream of CDS start codon and adjacent uORF stop codons. As mentioned above, EMF stage offers the most distinct uORF profile, making it the example of choice for translation repression analysis (see S6 Fig).

Maximum translation repression is enforced by uORFs overlapping the CDS start codon. More specifically, the window first including exclusively overlapping uORFs (identified by the blue box) also marks the minimum of Fig 11. Higher y-values on the left end side appear most likely due to virtual absence of extremely overlapping uORFs, prohibiting a linear correlation. Further upstream windows comprise more and more non-overlapping uORFs, gradually representing less translation repression. The x-values in Fig 11 between 0 nt and 55 nt are underlined by a red box, marking the range of non-overlapping uORF to CDSs distances of special interest. In this area, uORFs gradually shed their repressive potential with increasing separation from the CDS start, up to a distance of 80 nt, when the uORF ultimately loses capacity to affect translation [24,25]. Y-values gradually approximate and eventually reach 0 in the window including the last translation repressive uORFs (55 nt-80 nt), marking the loss of regulative potential (see Fig 11).

### Repression of housekeeping genes in EMF correlates with uORF status of transcripts

When investigating potentially uORF-mediated stage-specific gene regulation in *T*. *congolense*, EMF stage takes a special position. Compared to other life cycle stages, it presents the largest number of individual uORFs (see S6 Fig) and most obvious impact of uORFs on protein level (see Fig 11). Transcripts in EMFs have exceptionally long half-lives in *T*. *cruzi* [49], emphasizing the relevance of post-transcriptional gene regulation to limit the amount of protein translated from the stable mRNA stock. This concept of minimizing cell metabolism is considered to secure a pool of long living, non-dividing EMFs in the tsetse fly, which can return to proliferation when nutrition is provided [49]. In *T*. *congolense*, EMFs are the only immobile form throughout the life cycle, bound to the epithelium in the tsetse’s proboscis [2]. In this unfavorable environment, they are considered to maintain a population of trypanosomes in the vector, when infective motile MCFs are transmitted with the fly’s saliva to the mammalian bloodstream, where the major part of proliferation takes place [50].

Fig 9 and Fig 10 present uORFs, which are up-regulated from PCF to EMF and down-regulated from EMF to MCF, mostly followed by decreased and increased protein translation, respectively. The subset of uORFs undergoing both contrary changes consecutively (red scatters on the aforementioned Figures) potentially curb translation of certain genes in EMF and release repression, when trypanosome life cycle progresses to MCF. A total of 168 proximal or overlapping uORFs, belonging to 117 genes, meets these specifications. Gene ontology analysis of this gene subset against all 1,757 genes with available proteomic data summarizes the biological function of proteins, which are stage-specifically suppressed in EMF: significantly (p<0.05) enriched GO terms include translation related functions (aminoacyl-tRNA ligase activity), cell division (mitotic spindle elongation) and energy metabolism (pyridoxal kinase activity). The full list of enriched GO terms is provided in S1 Table. Notably, the accumulation of housekeeping genes in this list does not derive from a selection bias (due to the technical nature of mass spectrometry, genes with available proteomic data might be among the most extensively translated), because the GO analysis was performed internally of the subset, which is limited by availability of proteomic data. Therefore, the uORF-mediated suppression of basic cell metabolism in EMF might be a biological approach in *T*. *congolense* to secure a pool of trypanosomes in a low active metabolic state, surviving in the unfavorable environment of tsetse’s proboscis. More specifically, pyridoxal kinase is among the enriched GO terms (S3 Table), which is an essential element in trypanosomes sugar and amino acid metabolism [51]. Inhibition of pyridoxal kinase results in crucial growth restriction of *T*. *brucei*, rendering the enzyme a promising target for recent pharmacological research [51]. In a physiological fashion, the uORF-mediated repression of pyridoxal kinase could mark the transition to a latent survival stage of trypanosomes.

## Conclusions

The presented work for the first time annotates uORFs in significant part of the *T*. *congolense* genome and explores their impact on regulating the parasitic life cycle of the studied species. Jensen et al. [14] did not find conclusive hints for uORFs being involved in stage-specific gene regulation of *T*. *brucei*. Here we give evidence that the expression of selected genes is controlled by introducing and eliminating the 5’ UTR-embedded uORFs in the stage-specific transcriptome. Variation of commonly accepted features, which define the uORFs translation repressive potential, might help to fine tune protein expression. Employing the uORF stop to CDS start distance as an example, we demonstrated a continuous spectrum of translation repression, which can help meeting the restrictions of *T*. *congolense’s* varying environments (e.g. host vs. vector). Besides the established influential uORF features, this work additionally focuses on the uORFs’ amino acid and codon composition. The systemic adoption of metabolically costly and rare components throughout certain uORFs might impede their translation and ultimately diminish chances of downstream ribosome reinitiation. Going in line with this hypothesis, our data showed enrichment of optimized codons and amino acids upstream of extensively translated genes.

## Supporting information

S1 Fig Alternative trans-splicing sites influence uORF profiles. Numbers were assigned to each stop codon preceded by an upstream in-frame start codon (uAUG), increasing with increasing distance from the CDS start codon. The respective in-frame start codons are numbered in increasing order, the longest uORF’s start codon numbered with 1. If not stated otherwise, only the longest uORF of each stop codon was considered in this work for analyses. mRNAs transcribed from the same gene, but spliced at different sites, can contain distinct uORFs. In the presented example, mRNA deriving from trans-splicing site A harbors two uORFs in its 5’ UTR, *uORF-2-1* and *uORF-1-1* being the longest variants. Alternative mRNA deriving from splice site B comprises two uORFs (longest uORFs are *uORF-1-1* and *uORF-2-2*) and mature transcripts spliced at site C only contain one uORF (longest uORF is *uORF-1-1*). Only 68 uORFs in total show different longest variants throughout the different life cycle stages (see S1 Data).
(PNG)

S2 Fig CAI and ASI are biased by length in short sequences (< 60 nt). (**A**) CAI was first employed by Sharp & Li (1987) as a model for optimization of codons throughout CDSs. (**B**) ASI was introduced above as a standard for deviation of amino acid profiles. Both concepts perform best for long sequences, when the influence of each component does not reach an overwhelming threshold on the calculation. This point is situated around a sequence length of 60 nt (dashed line), which divides the uORFs into a length dependent and generally length independent subset (see r values). 60 nt sequence length was chosen as an arbitrary cutoff for all CAI and ASI analyses throughout this work.
(TIFF)

S3 Fig Characteristics of *T*. *congolense* uORFs. (**A**) uORF length varies between 6 nt and 4,518 nt, median uORF length (dashed line) equals to 51.0 nt (Q1 = 24 nt, Q2 = 105 nt, n = 18,511). (**B**) The distance from uORF stop codon to upstream CDS start codon varies between -4,257 nt and 1,978 nt. Median is 447 nt (Q1 = 183.5 nt, Q3 = 782.0 nt). Negative values represent uORFs overlapping the CDS start codon (uORF stop situated downstream of CDS start codon). (**C**) To measure the codon usage bias of *T*. *congolense* uORFs, the normalized usage ratio of the most frequently used codon was divided by the least preferred in each subset of synonymous codons. *T*. *congolense* uORFs show a distinct codon bias. (**D**) Amino acid usage frequencies differ significantly from the expected value 1/20 (two-sided binomial test, p<0.05). The most frequently used amino acid leucine (9.9% of uORF sequence) also shows the largest codon bias (the preferred codon is used 3.15 fold compared to the rarest codon).
(TIFF)

S4 Fig uORF and non-uORF sequences of 5' UTR show preference for different amino acids and codons. (**A**) The difference of amino acid usage between uORFs and non-uORF 5’ UTR is displayed by the log2 ratios of amino acid usage. (**B**) The bias of codon usage between uORFs and non-uORFs is shown by the sum of the log2 of normalized codon ratios for each subgroup of synonymous codons. For instance, aspartic acid (D) is presented 1.19 fold more frequent by “GAT” and used 1.59 fold more frequent in uORFs as compared to non-uORF 5’ UTR. The plot does not show calculations of methionine, because by definition non-uORF 5' UTR does not contain start codons.
(TIFF)

S5 Fig Characteristics of *T*. *congolense* CDSs and UTRs. (**A**) The length of protein coding CDSs with annotated 5’ UTR varies in range from 78 nt to 18,873 nt. Median length (dashed line) is 1,137.0 nt (Q1 = 714.0 nt, Q3 = 1,758.0 nt). (**B**) Among genes that show at least one uORF, the maximum amount of uORFs varies in a range from one to 34 per gene. Median number is six uORFs per gene (Q1 = 1.0, Q3 = 11.0). (**C**) Codon usage bias is presented by the ratio of the frequencies of the most frequently used codon divided by the rarest. Leucine (L) shows the most biased codons (“CTG” is used 3.04 times as often as “CTA”). (**D**) Furthermore, leucine (L) is the most common amino acid and makes up almost 10% of *T*. *congolense* CDSs. (**E**) Length of annotated 5’ UTRs varies in range from 0 nt to 2,000 nt, with a median length (dashed line) of 127.0 nt. (**F**) 5’ UTR length correlates with the number of harbored uORFs (r = 0.88), i.e. larger transcript leaders generally accommodate a greater number of uORFs.
(TIFF)

S6 Fig Venn diagram of longest present uORFs throughout the life cycle. A total of 18,511 uORFs is distributed among the life cycle stages of *T*. *congolense*. The biggest amount of uORFs is shared by all four stages (62.2%), while some uORFs are unique to certain stages. EMF has a significantly higher number of unique uORFs (absolute 1,026 uORFs), while all other stages only express around 400 unique uORFs (chi-squared test, p<0.05).
(PNG)

S7 Fig uORFs and CDSs prefer different codons. Codon usage frequencies of CDSs were divided by the respective frequencies in uORFs and the log2 of the ratio was plotted. Positive values represent codons preferred by CDSs and vice versa. The codon usage bias between CDSs and uORFs is significant for 59 out of 64 codons (chi-squared test, p<0.05, five codons marked with * are not significantly biased).
(PNG)

S8 Fig Codon usage in uORFs of highly translated genes is optimized. For each amino acid the normalized codon usage frequencies were determined throughout uORFs contained in extensively translated genes and the complementary subsets (see Fig 5). Subsequently an optimized codon profile was extracted from particularly these CDSs, which scored the top 10% of CAI. The sum of log2 of the normalized codon profiles of uORFs in the above mentioned gene subsets and the best optimized CDSs was calculated. A larger y-value represents a relatively great divergence from the optimized CDS codon usage. Throughout most tandem and top CAI uORFs, the codon profile is more related to top CAI CDSs in all groups of synonymous codons. Adoption of optimized codons in uORFs might be a strategy to positively influence mRNA stability in the mentioned subsets. Codon profile in non-proximal uORFs of the subset of proteome available genes does not show a distinct bias towards or against optimized codons (also compare CAIs in Table 1).
(TIFF)

S9 Fig Completion of Fig 1 containing all life cycle stages. (**A**) all uORFs, (**B**) proximal uORFs. For detailed description please see Fig 1.
(TIFF)

S10 Fig Completion of Fig 2 containing all life cycle stages. (**A**) all uORFs, (**B**) proximal uORFs. For detailed description please see Fig 3.
(TIFF)

S1 Text Tandem genes in *T*. *congolense*. (DOCX)

S1 Table Significantly enriched GO terms among tandem amplified genes, excluding BIN chromosome (p<0.05). (DOCX)

S2 Table Significantly enriched GO terms among top 100 CAI genes (p<0.05). (DOCX)

S3 Table Significantly enriched GO terms among genes with up regulated and consecutively down regulated uORFs from PCF to EMF and EMF to MCF, respectively, paired with decreased and increased protein translation, respectively (p<0.05). (DOCX)

S1 Data Basic uORF and CDS data. Column names are included as the first row. uORF Illumina reads for redundant uORFs, which are fully contained in the stage specific longest uORF, are defined as 0. Stage specific splice sites are presented in the format “identifier, start coordinate, end coordinate, # of mapped Illumina reads”. The file comprises of 39494 entries– 31,149 for uORFs and 8,345 CDSs.
(TXT)

S2 Data Amino acid and codon profiles of uORFs and CDSs. Column names are included as the first row.
(TXT)

S3 Data Amino acid and codon profiles of non-uORF 5’ UTR, first frame. Column names are included as the first row.
(TXT)

S4 Data Amino acid and codon profiles of non-uORF 5’ UTR, second frame. Column names are included as the first row.
(TXT)

S5 Data Amino acid and codon profiles of non-uORF 5’ UTR, third frame. Column names are included as the first row.
(TXT)

S6 Data Annotations of 5’UTRs in GFF3 format for TriTrypDB 25 genes. Information on number of mapped tags per trans-splicing site is provided in as Note = N, where N is the number of tags.
(GFF)

S7 Data Gene expression profiles in FPKMs as calculated by Cufflinks software for T. congolense genes annotated in TriTrypDB 25 database. Values derive from the RNA-seq experiments of all four life cycle stages of *T*. *congolense* with five biological replicates each. Jąkalski et al. (unpublished)
(XLSX)
