# Supplementary figures and images for: Life cycle adapted upstream open reading frames (uORFs) in Trypanosoma congolense: A post-transcriptional approach to accurate gene regulation
Source: PLoS One. 2018 Aug 9;13(8):e0201461. doi: 10.1371/journal.pone.0201461 (PMC6084854; doi:10.1371/journal.pone.0201461)

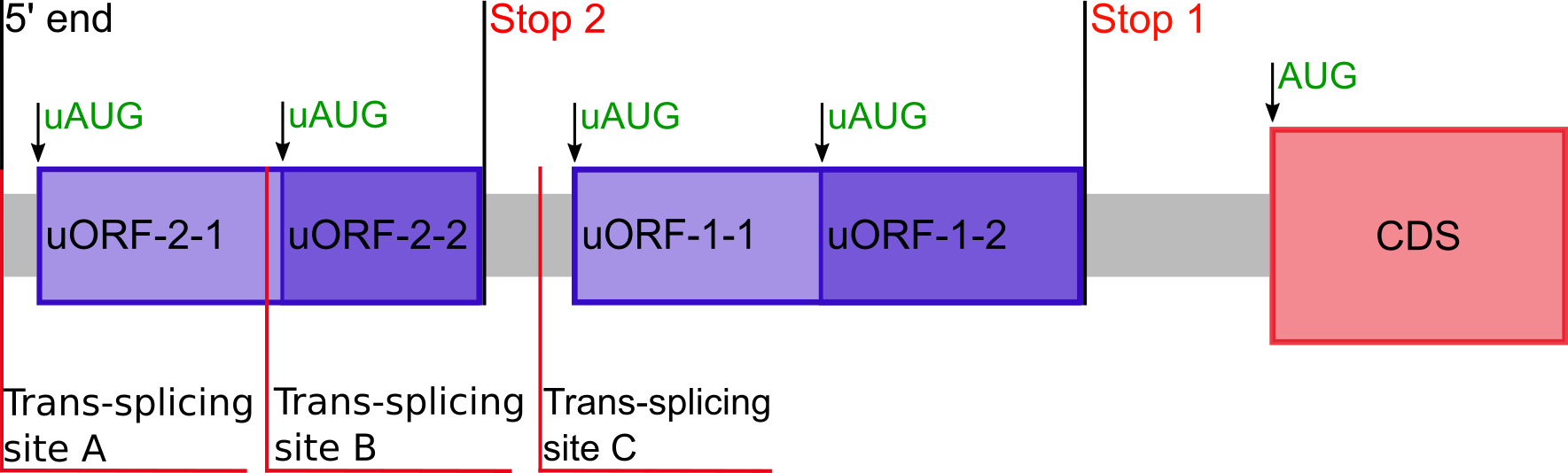

Supplement: S1 Fig — Numbers were assigned to each stop codon preceded by an upstream in-frame start codon (uAUG), increasing with increasing distance from the CDS start codon. The respective in-frame start codons are numbered in increasing order, the longest uORF’s start codon numbered with 1. If not stated otherwise, only the longest uORF of each stop codon was considered in this work for analyses. mRNAs transcribed from the same gene, but spliced at different sites, can contain distinct uORFs. In the presented example, mRNA deriving from trans-splicing site A harbors two uORFs in its 5’ UTR, uORF-2-1 and uORF-1-1 being the longest variants. Alternative mRNA deriving from splice site B comprises two uORFs (longest uORFs are uORF-1-1 and uORF-2-2) and mature transcripts spliced at site C only contain one uORF (longest uORF is uORF-1-1). Only 68 uORFs in total show different longest variants throughout the different life cycle stages (see S1 Data). (PNG) [file pone.0201461.s001.png]

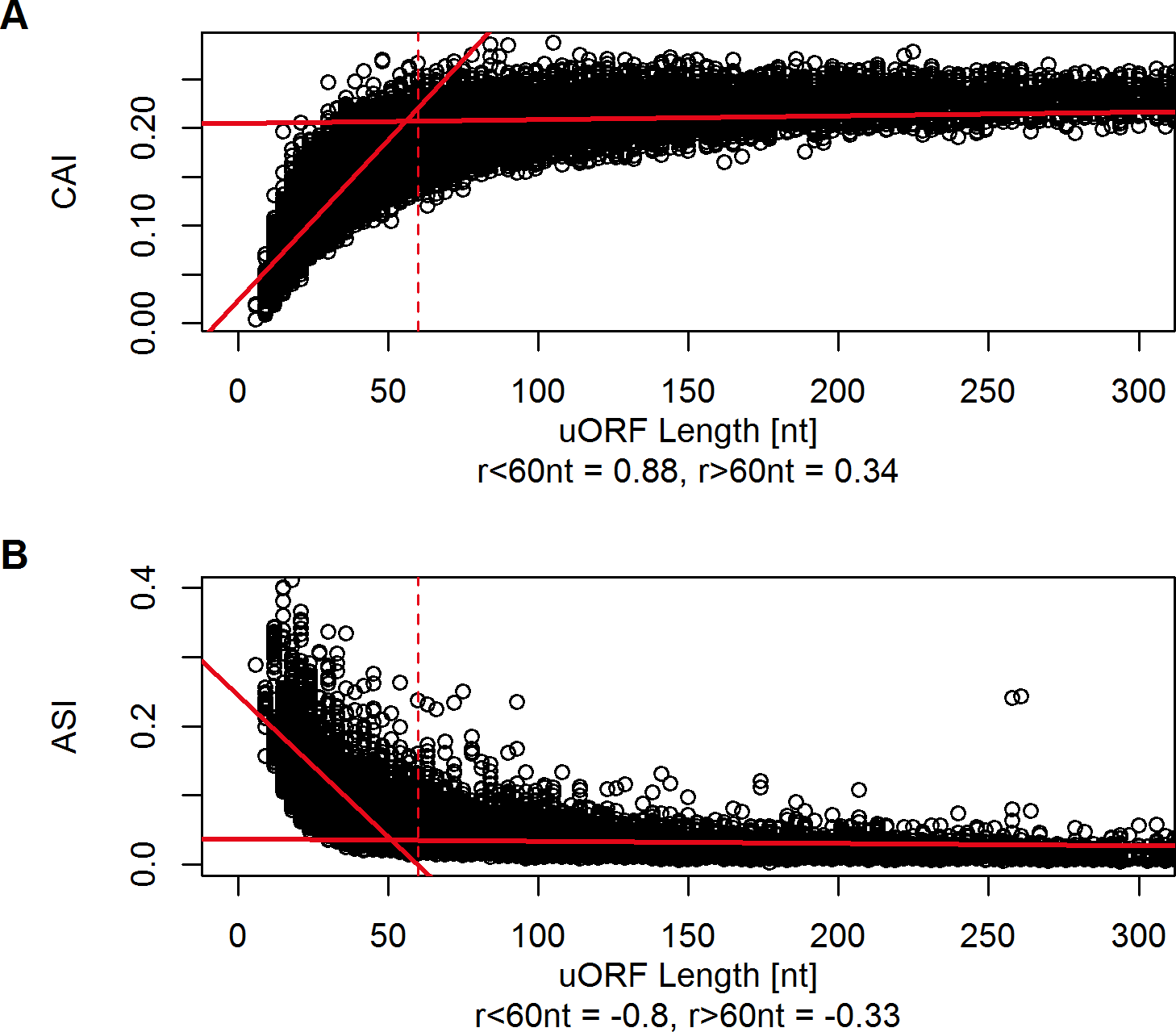

Supplement: S2 Fig — (A) CAI was first employed by Sharp & Li (1987) as a model for optimization of codons throughout CDSs. (B) ASI was introduced above as a standard for deviation of amino acid profiles. Both concepts perform best for long sequences, when the influence of each component does not reach an overwhelming threshold on the calculation. This point is situated around a sequence length of 60 nt (dashed line), which divides the uORFs into a length dependent and generally length independent subset (see r values). 60 nt sequence length was chosen as an arbitrary cutoff for all CAI and ASI analyses throughout this work. (TIFF) [file pone.0201461.s002.tiff]

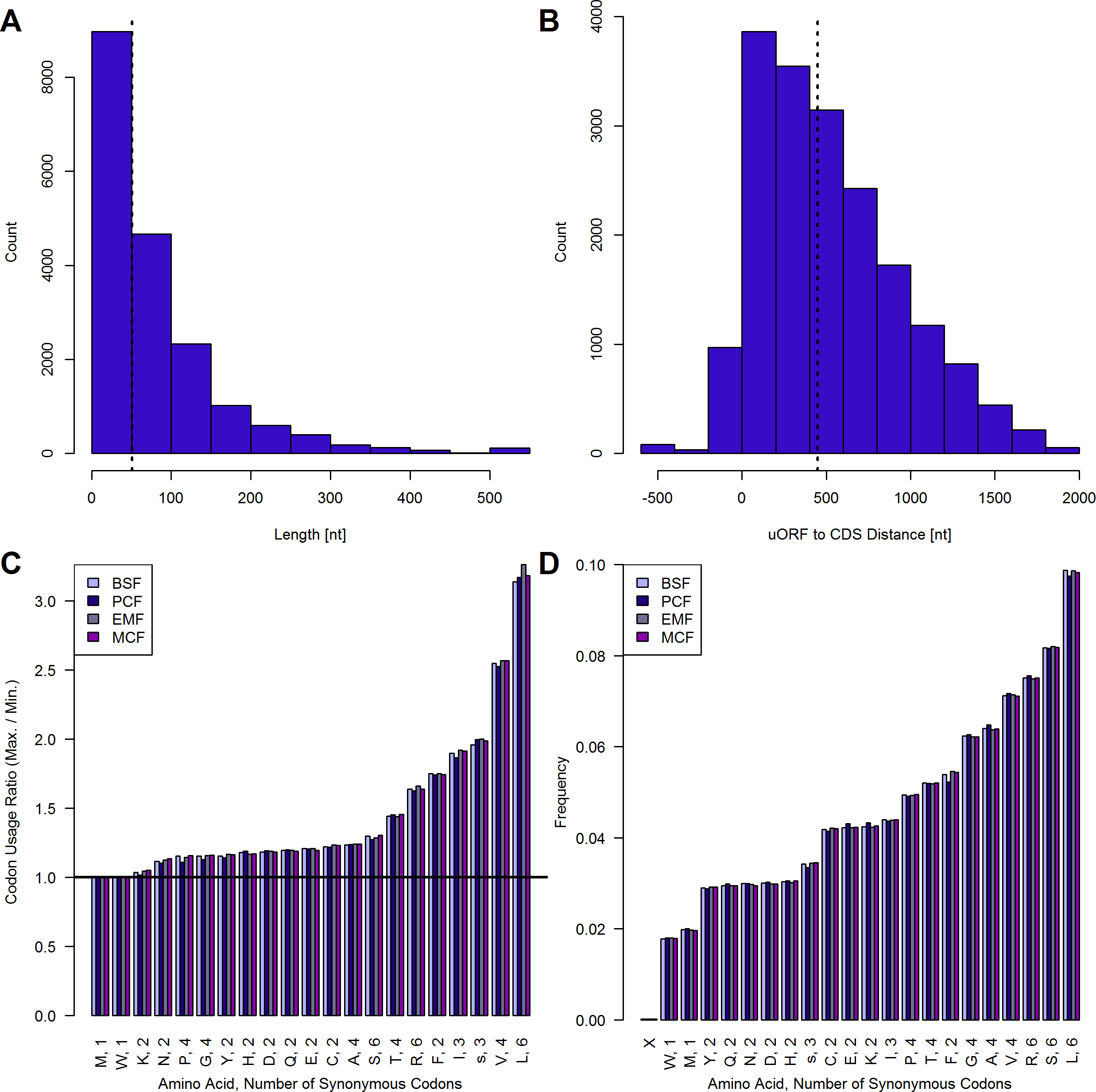

Supplement: S3 Fig — (A) uORF length varies between 6 nt and 4,518 nt, median uORF length (dashed line) equals to 51.0 nt (Q1 = 24 nt, Q2 = 105 nt, n = 18,511). (B) The distance from uORF stop codon to upstream CDS start codon varies between -4,257 nt and 1,978 nt. Median is 447 nt (Q1 = 183.5 nt, Q3 = 782.0 nt). Negative values represent uORFs overlapping the CDS start codon (uORF stop situated downstream of CDS start codon). (C) To measure the codon usage bias of T. congolense uORFs, the normalized usage ratio of the most frequently used codon was divided by the least preferred in each subset of synonymous codons. T. congolense uORFs show a distinct codon bias. (D) Amino acid usage frequencies differ significantly from the expected value 1/20 (two-sided binomial test, p<0.05). The most frequently used amino acid leucine (9.9% of uORF sequence) also shows the largest codon bias (the preferred codon is used 3.15 fold compared to the rarest codon). (TIFF) [file pone.0201461.s003.tiff]

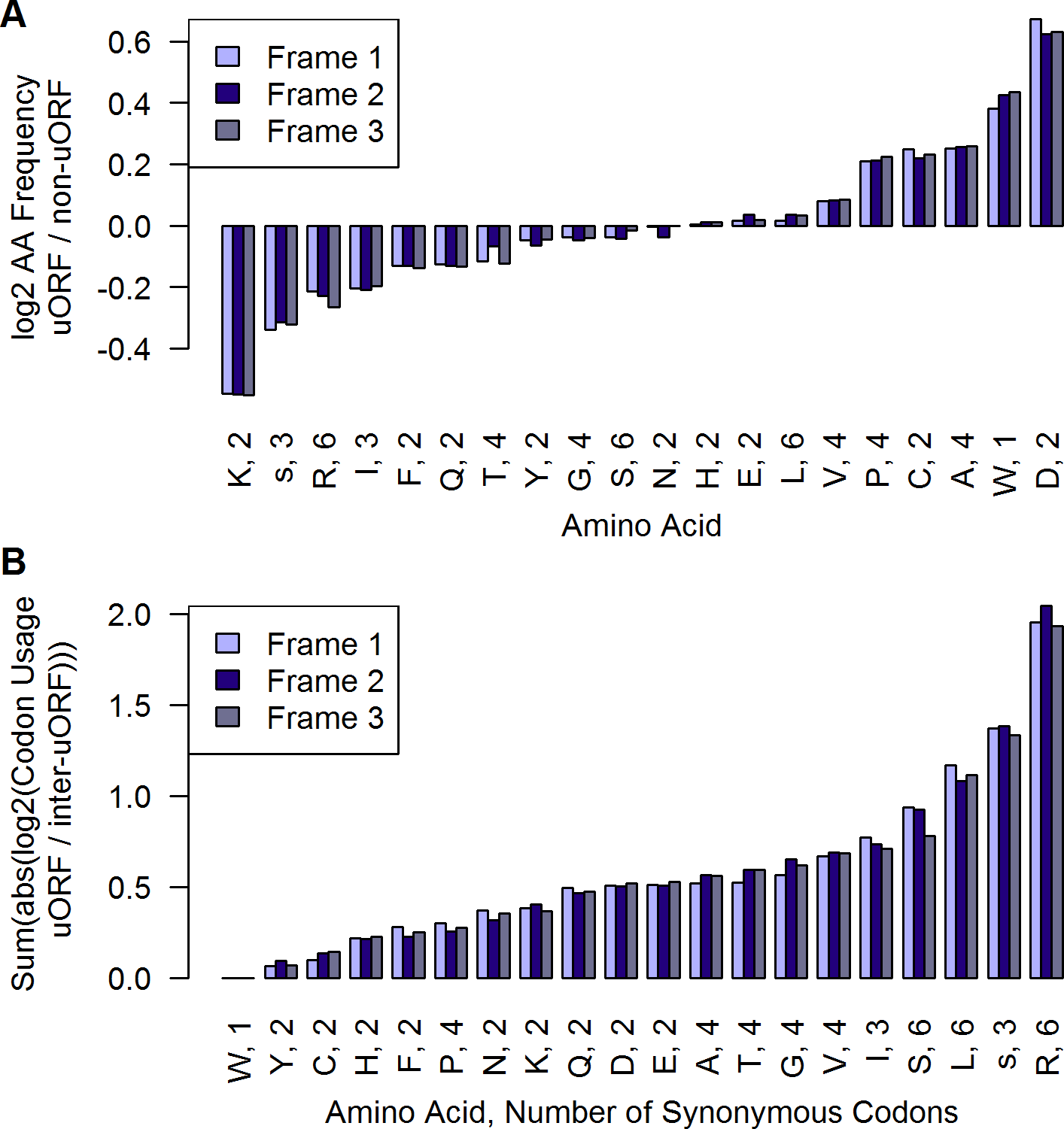

Supplement: S4 Fig — (A) The difference of amino acid usage between uORFs and non-uORF 5’ UTR is displayed by the log2 ratios of amino acid usage. (B) The bias of codon usage between uORFs and non-uORFs is shown by the sum of the log2 of normalized codon ratios for each subgroup of synonymous codons. For instance, aspartic acid (D) is presented 1.19 fold more frequent by “GAT” and used 1.59 fold more frequent in uORFs as compared to non-uORF 5’ UTR. The plot does not show calculations of methionine, because by definition non-uORF 5' UTR does not contain start codons. (TIFF) [file pone.0201461.s004.tiff]

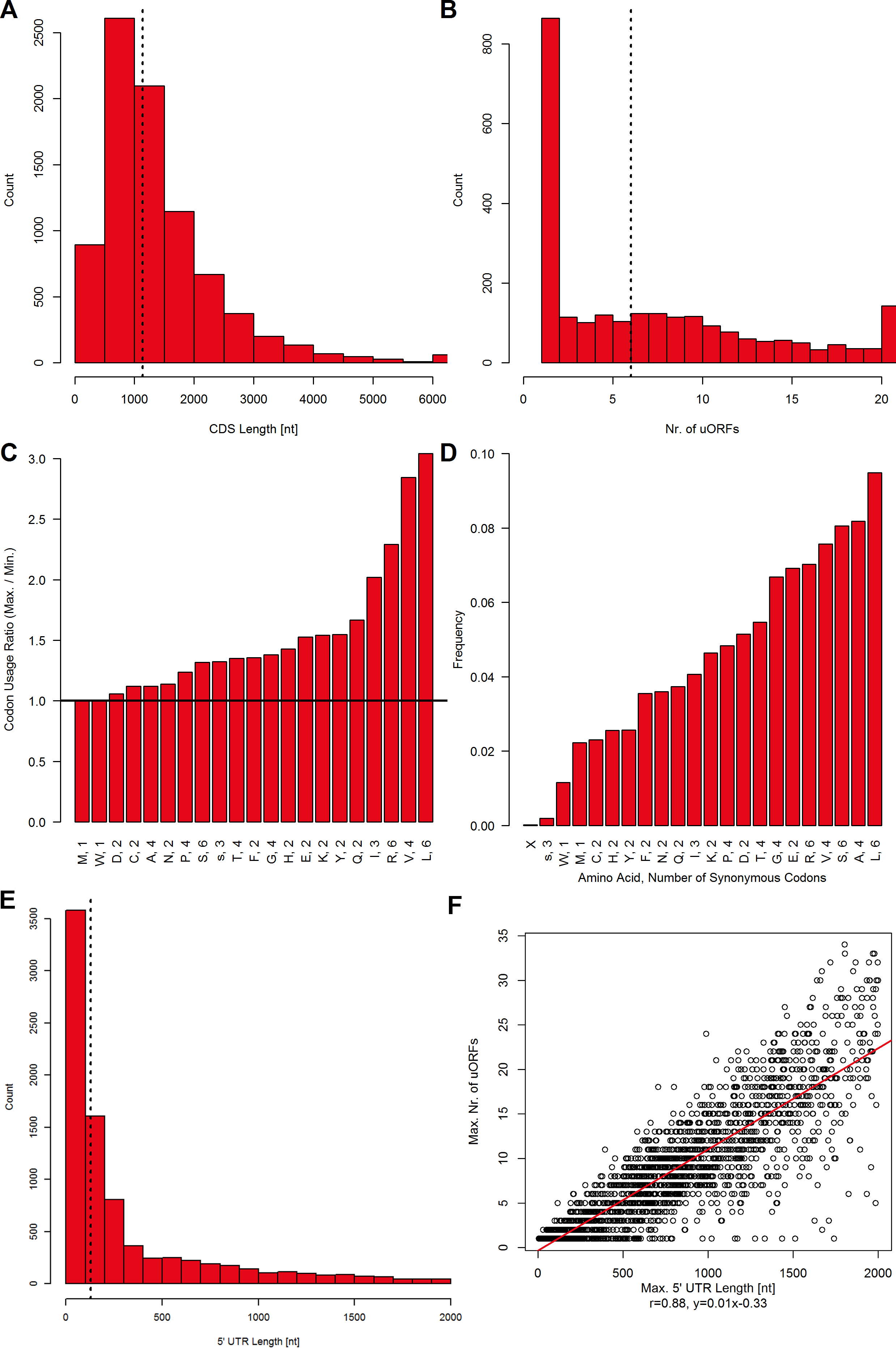

Supplement: S5 Fig — (A) The length of protein coding CDSs with annotated 5’ UTR varies in range from 78 nt to 18,873 nt. Median length (dashed line) is 1,137.0 nt (Q1 = 714.0 nt, Q3 = 1,758.0 nt). (B) Among genes that show at least one uORF, the maximum amount of uORFs varies in a range from one to 34 per gene. Median number is six uORFs per gene (Q1 = 1.0, Q3 = 11.0). (C) Codon usage bias is presented by the ratio of the frequencies of the most frequently used codon divided by the rarest. Leucine (L) shows the most biased codons (“CTG” is used 3.04 times as often as “CTA”). (D) Furthermore, leucine (L) is the most common amino acid and makes up almost 10% of T. congolense CDSs. (E) Length of annotated 5’ UTRs varies in range from 0 nt to 2,000 nt, with a median length (dashed line) of 127.0 nt. (F) 5’ UTR length correlates with the number of harbored uORFs (r = 0.88), i.e. larger transcript leaders generally accommodate a greater number of uORFs. (TIFF) [file pone.0201461.s005.tiff]

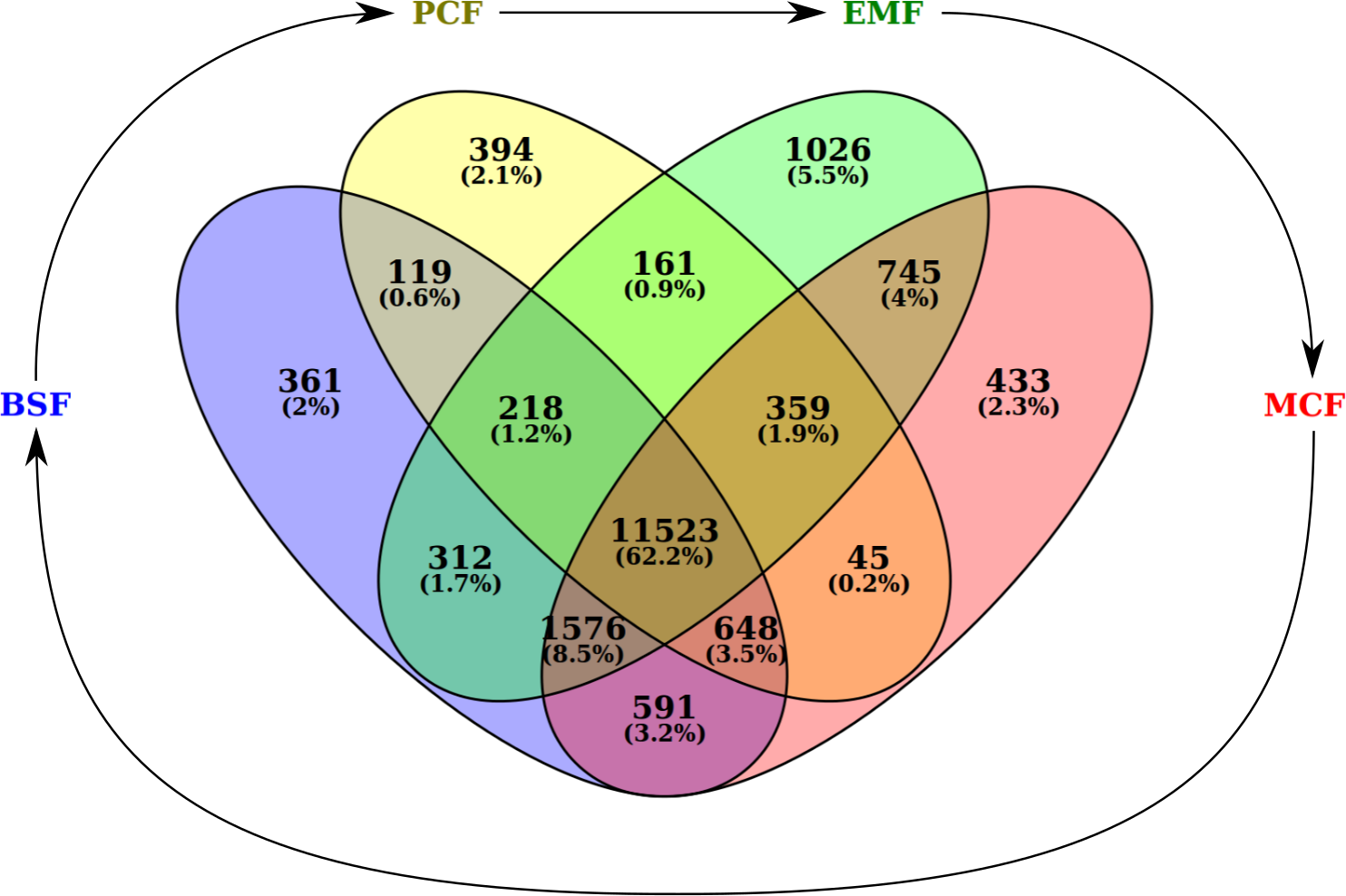

Supplement: S6 Fig — A total of 18,511 uORFs is distributed among the life cycle stages of T. congolense. The biggest amount of uORFs is shared by all four stages (62.2%), while some uORFs are unique to certain stages. EMF has a significantly higher number of unique uORFs (absolute 1,026 uORFs), while all other stages only express around 400 unique uORFs (chi-squared test, p<0.05). (PNG) [file pone.0201461.s006.png]

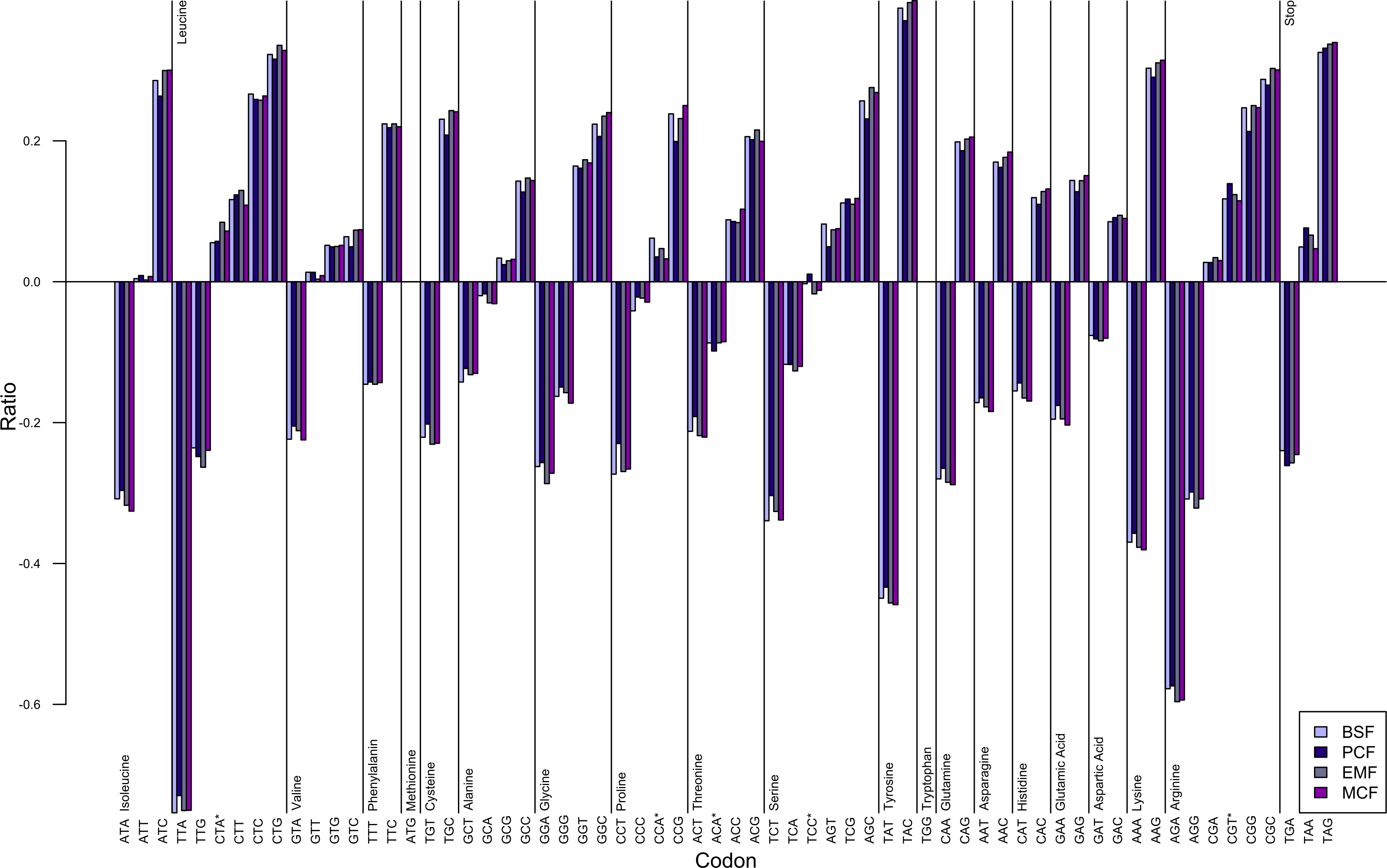

Supplement: S7 Fig — Codon usage frequencies of CDSs were divided by the respective frequencies in uORFs and the log2 of the ratio was plotted. Positive values represent codons preferred by CDSs and vice versa. The codon usage bias between CDSs and uORFs is significant for 59 out of 64 codons (chi-squared test, p<0.05, five codons marked with * are not significantly biased). (PNG) [file pone.0201461.s007.png]

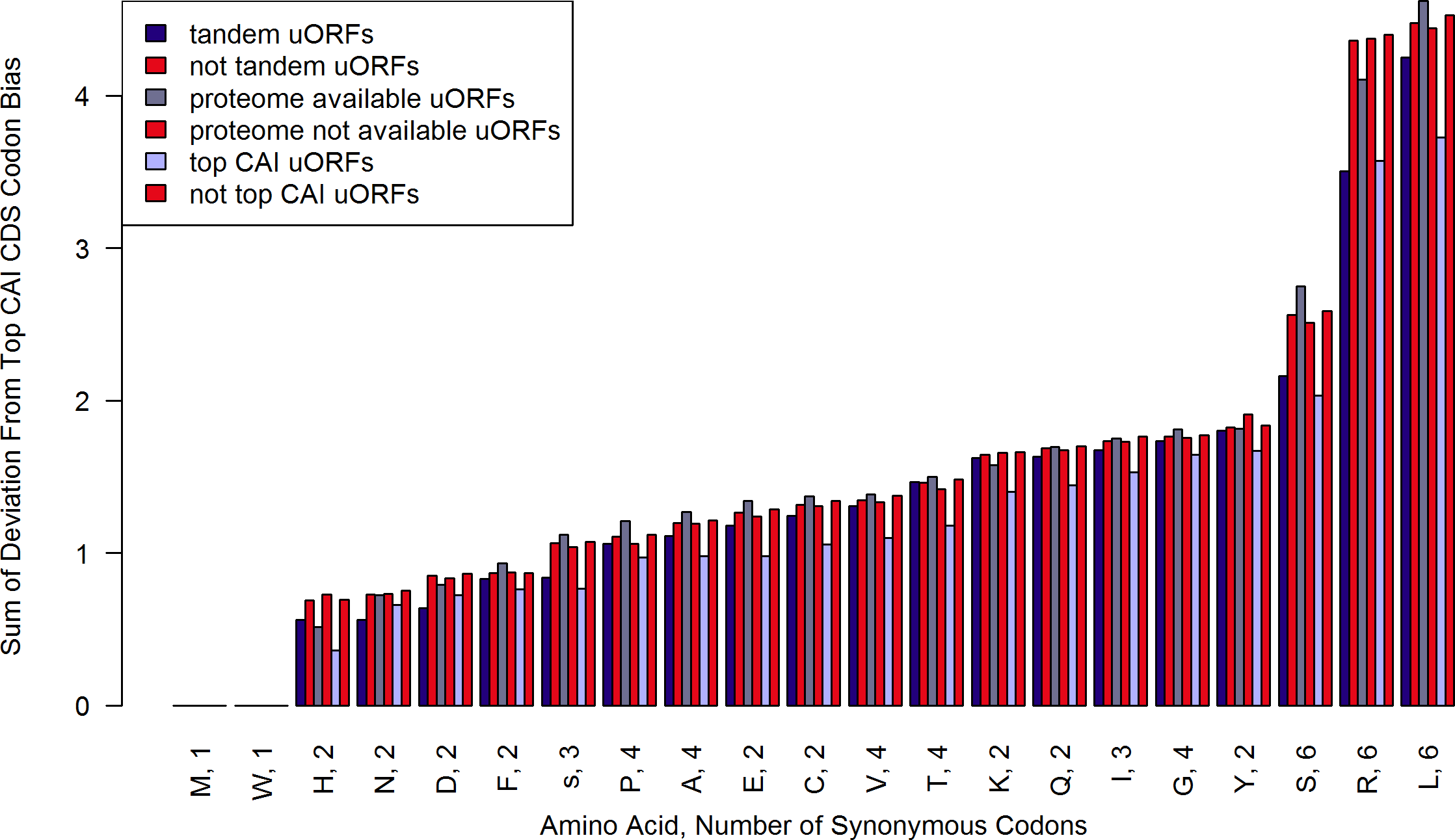

Supplement: S8 Fig — For each amino acid the normalized codon usage frequencies were determined throughout uORFs contained in extensively translated genes and the complementary subsets (see Fig 5). Subsequently an optimized codon profile was extracted from particularly these CDSs, which scored the top 10% of CAI. The sum of log2 of the normalized codon profiles of uORFs in the above mentioned gene subsets and the best optimized CDSs was calculated. A larger y-value represents a relatively great divergence from the optimized CDS codon usage. Throughout most tandem and top CAI uORFs, the codon profile is more related to top CAI CDSs in all groups of synonymous codons. Adoption of optimized codons in uORFs might be a strategy to positively influence mRNA stability in the mentioned subsets. Codon profile in non-proximal uORFs of the subset of proteome available genes does not show a distinct bias towards or against optimized codons (also compare CAIs in Table 1). (TIFF) [file pone.0201461.s008.tiff]

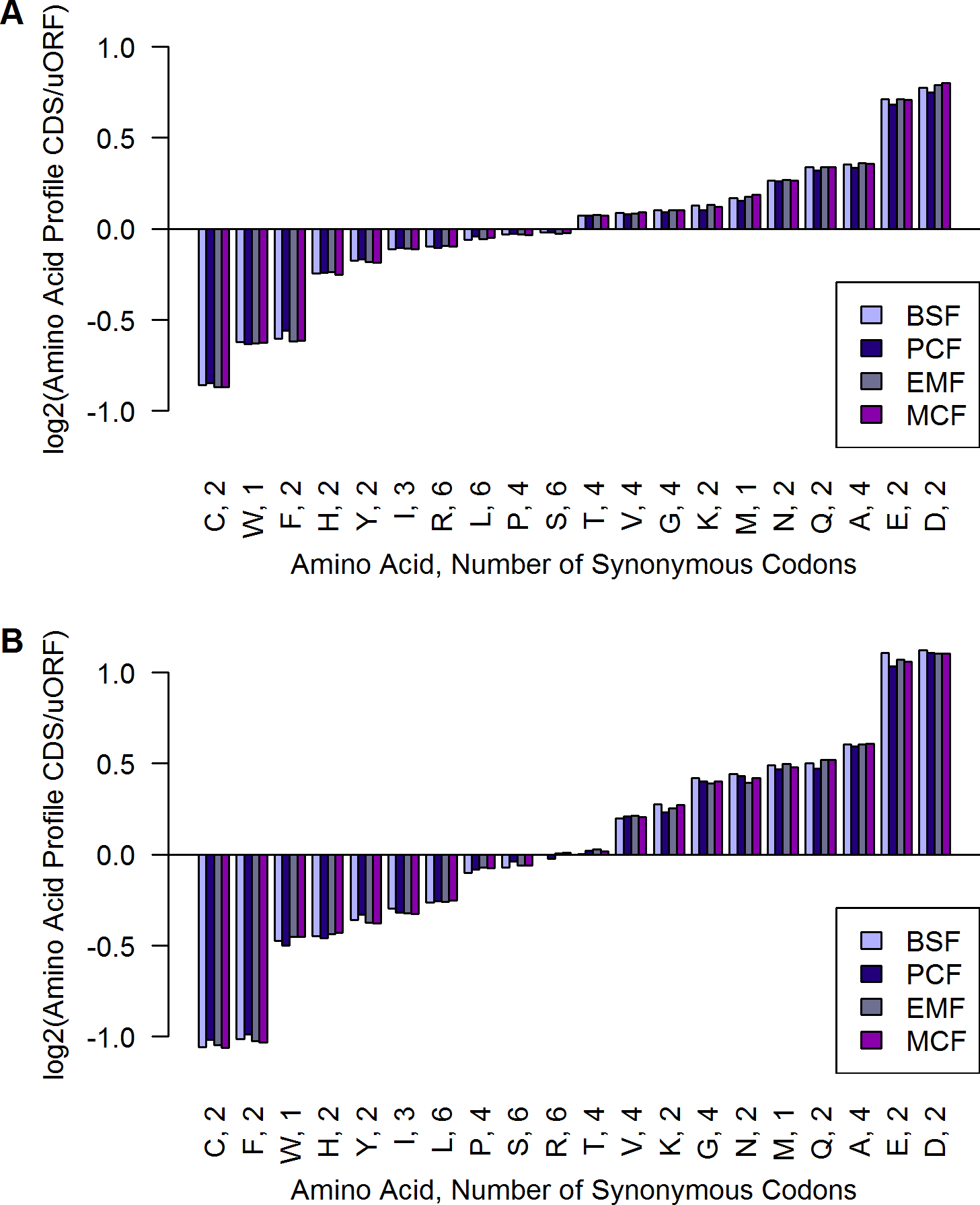

Supplement: S9 Fig — (A) all uORFs, (B) proximal uORFs. For detailed description please see Fig 1. (TIFF) [file pone.0201461.s009.tiff]

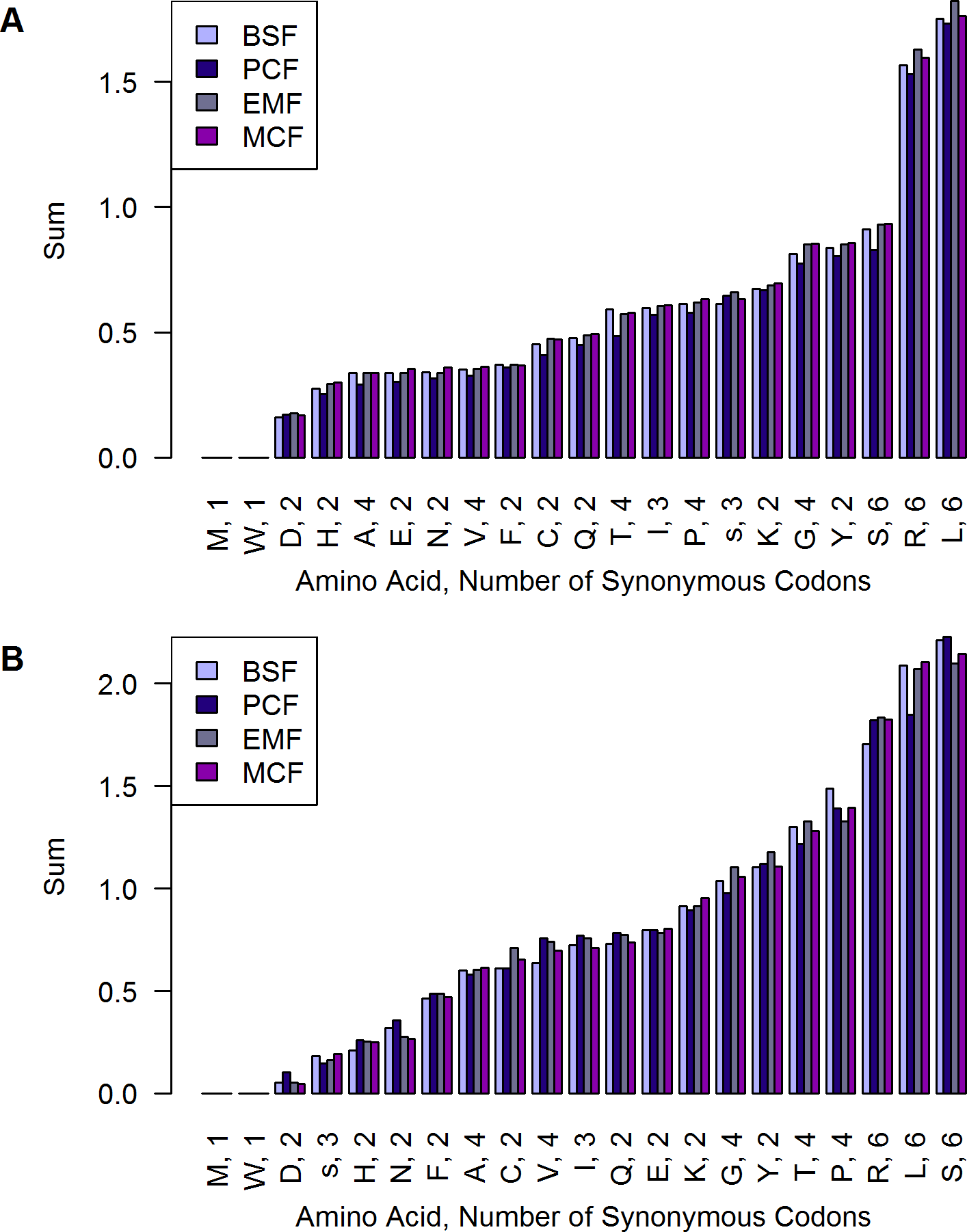

Supplement: S10 Fig — (A) all uORFs, (B) proximal uORFs. For detailed description please see Fig 3. (TIFF) [file pone.0201461.s010.tiff]
