# Supplementary material for: Life cycle adapted upstream open reading frames (uORFs) in Trypanosoma congolense: A post-transcriptional approach to accurate gene regulation
Source: PLoS One. 2018 Aug 9;13(8):e0201461. doi: 10.1371/journal.pone.0201461 (PMC6084854; doi:10.1371/journal.pone.0201461)
Supplement: S1 Text — (DOCX) [file pone.0201461.s011.docx]

**S1 Text:** Tandem genes in *T. congolense*.

Throughout the subset of genes with predicted 5’ UTR, a total of 609 tandem amplified genes were computationally annotated, containing 19 TGAs with at least four gene copies (see S13 Data). Jackson et al. [1] identified 47 TGAs comprising at least four elements in *T. brucei* genome. Assuming vast sequence similarity of the two trypanosome species *T. brucei* and *T. congolense*, Jackson’s work can be related to, to verify the method of finding TGAs employed in this work. Tandem analysis of the BIN pseudochromosome reveals that the difference of 28 TGAs compared to *T. brucei* was most likely lost during the scaffolding and annotation process of the BIN pseudochromosome. Due to the unordered nature of this artificial chromosome, and the possibility of false positively identified tandems, TGAs on the BIN pseudochromosome were not considered for further calculations. In order to validate the biological significance of the annotated tandem genes, a gene ontology analysis was performed. Significantly enriched gene ontologies include genes associated with translation (structural constituents of ribosome, elongation) and energy metabolism (glycolysis, gluconeogenesis, fatty acid metabolism, ATP biosynthesis), which ensure basic functioning of the cell. These findings are in line with the work by Horn et al. [2], suggesting tandem amplification as an indicator for expression level in *T. brucei*. The full list of enriched gene ontologies is attached in S12 Table.

**REFERENCES:**

1. Jackson AP, Miller W, Makova K, Nekrutenko A, Hardison R, Bentley S, et al. Tandem gene arrays in Trypanosoma brucei: Comparative phylogenomic analysis of duplicate sequence variation. BMC Evol Biol. BioMed Central; 2007;7: 54. doi:10.1186/1471-2148-7-54

2. Horn D, Palenchar J, Bellofatto V, El-Sayed N, Myler P, Blandin G, et al. Codon usage suggests that translational selection has a major impact on protein expression in trypanosomatids. BMC Genomics. BioMed Central; 2008;9: 2. doi:10.1186/1471-2164-9-2
