# Supplementary material for: Life cycle adapted upstream open reading frames (uORFs) in Trypanosoma congolense: A post-transcriptional approach to accurate gene regulation
Source: PLoS One. 2018 Aug 9;13(8):e0201461. doi: 10.1371/journal.pone.0201461 (PMC6084854; doi:10.1371/journal.pone.0201461)
Supplement: S1 Table — (DOCX) [file pone.0201461.s012.docx]

**S1 Table:** Significantly enriched GO terms among tandem amplified genes, excluding BIN chromosome (p<0.05).

| GO term | p-value | molecular function / biological process |
| --- | --- | --- |
| GO:0046982 | 6.4E-08 | protein heterodimerization activity |
| GO:0006334 | 3.0E-07 | nucleosome assembly |
| GO:0003735 | 5.1E-06 | structural constituent of ribosome |
| GO:0006412 | 1.9E-05 | translation |
| GO:0006096 | 2.3E-05 | glycolytic process |
| GO:0003676 | 2.7E-05 | nucleic acid binding |
| GO:0004618 | 9.7E-04 | phosphoglycerate kinase activity |
| GO:0016246 | 1.4E-03 | RNA interference |
| GO:0030422 | 1.4E-03 | production of siRNA involved in RNA interference |
| GO:0004321 | 1.4E-03 | fatty-acyl-CoA synthase activity |
| GO:0004576 | 1.4E-03 | oligosaccharyl transferase activity |
| GO:0004612 | 1.4E-03 | phosphoenolpyruvate carboxykinase (ATP) activity |
| GO:0006650 | 1.4E-03 | glycerophospholipid metabolic process |
| GO:0047396 | 1.4E-03 | glycosylphosphatidylinositol diacylglycerol-lyase activity |
| GO:0004332 | 1.4E-03 | fructose-bisphosphate aldolase activity |
| GO:0045041 | 1.4E-03 | protein import into mitochondrial intermembrane space |
| GO:0008017 | 2.3E-03 | microtubule binding |
| GO:0005524 | 3.1E-03 | ATP binding |
| GO:0006414 | 3.2E-03 | translational elongation |
| GO:0006486 | 4.1E-03 | protein glycosylation |
| GO:0004611 | 4.1E-03 | phosphoenolpyruvate carboxykinase activity |
| GO:0017076 | 4.1E-03 | purine nucleotide binding |
| GO:0022891 | 4.1E-03 | substrate-specific transmembrane transporter activity |
| GO:0004571 | 4.1E-03 | mannosyl-oligosaccharide 1,2-alpha-mannosidase activity |
| GO:0016209 | 4.1E-03 | antioxidant activity |
| GO:0051920 | 4.1E-03 | peroxiredoxin activity |
| GO:0006812 | 4.9E-03 | cation transport |
| GO:0007018 | 8.0E-03 | microtubule-based movement |
| GO:0004713 | 8.1E-03 | protein tyrosine kinase activity |
| GO:0006754 | 8.1E-03 | ATP biosynthetic process |
| GO:0043022 | 8.1E-03 | ribosome binding |
| GO:0006094 | 8.1E-03 | gluconeogenesis |
| GO:0022857 | 8.1E-03 | transmembrane transporter activity |
| GO:0004797 | 8.1E-03 | thymidine kinase activity |
| GO:0003972 | 8.1E-03 | RNA ligase (ATP) activity |
| GO:0006388 | 8.1E-03 | tRNA splicing, via endonucleolytic cleavage and ligation |
| GO:0016972 | 8.1E-03 | thiol oxidase activity |
| GO:0006468 | 9.9E-03 | protein phosphorylation |
| GO:0004672 | 9.9E-03 | protein kinase activity |
| GO:0003777 | 1.1E-02 | microtubule motor activity |
| GO:0005509 | 1.3E-02 | calcium ion binding |
| GO:0009168 | 1.3E-02 | purine ribonucleoside monophosphate biosynthetic process |
| GO:0019239 | 1.3E-02 | deaminase activity |
| GO:0006631 | 1.3E-02 | fatty acid metabolic process |
| GO:0004629 | 1.9E-02 | phospholipase C activity |
| GO:0005516 | 1.9E-02 | calmodulin binding |
| GO:0050660 | 2.8E-02 | flavin adenine dinucleotide binding |
| GO:0003746 | 3.4E-02 | translation elongation factor activity |
| GO:0008645 | 3.8E-02 | hexose transport |
| GO:0015149 | 3.8E-02 | hexose transmembrane transporter activity |
| GO:0000082 | 3.8E-02 | G1/S transition of mitotic cell cycle |
| GO:0004396 | 3.8E-02 | hexokinase activity |
| GO:0045454 | 4.1E-02 | cell redox homeostasis |
| GO:0051276 | 4.3E-02 | chromosome organization |
| GO:0008081 | 4.3E-02 | phosphoric diester hydrolase activity |
| GO:0003729 | 4.3E-02 | mRNA binding |
