# Supplementary material for: Life cycle adapted upstream open reading frames (uORFs) in Trypanosoma congolense: A post-transcriptional approach to accurate gene regulation
Source: PLoS One. 2018 Aug 9;13(8):e0201461. doi: 10.1371/journal.pone.0201461 (PMC6084854; doi:10.1371/journal.pone.0201461)
Supplement: S2 Table — (DOCX) [file pone.0201461.s013.docx]

**S2 Table:** Significantly enriched GO terms among top 100 CAI genes (p<0.05).

| GO term | p-value | molecular function / biological process |
| --- | --- | --- |
| GO:0005509 | 2.9E-05 | calcium ion binding |
| GO:0004618 | 3.7E-05 | phosphoglycerate kinase activity |
| GO:0004612 | 1.5E-04 | phosphoenolpyruvate carboxykinase (ATP) activity |
| GO:0003735 | 2.2E-04 | structural constituent of ribosome |
| GO:0006412 | 3.0E-04 | translation |
| GO:0004611 | 3.9E-04 | phosphoenolpyruvate carboxykinase activity |
| GO:0017076 | 3.9E-04 | purine nucleotide binding |
| GO:0006094 | 7.5E-04 | gluconeogenesis |
| GO:0006096 | 1.2E-03 | glycolytic process |
| GO:0005516 | 1.6E-03 | calmodulin binding |
| GO:0004427 | 3.5E-03 | inorganic diphosphatase activity |
| GO:0051082 | 3.7E-03 | unfolded protein binding |
| GO:0051258 | 7.7E-03 | protein polymerization |
| GO:0044267 | 1.3E-02 | cellular protein metabolic process |
| GO:0032054 | 1.3E-02 | ciliary basal body duplication |
| GO:0008097 | 1.3E-02 | 5S rRNA binding |
| GO:0043056 | 2.0E-02 | forward locomotion |
| GO:0006457 | 2.0E-02 | protein folding |
| GO:0006928 | 2.4E-02 | movement of cell or subcellular component |
| GO:0051539 | 2.4E-02 | 4 iron, 4 sulfur cluster binding |
| GO:0000910 | 2.6E-02 | cytokinesis |
| GO:0042255 | 2.6E-02 | ribosome assembly |
| GO:0044782 | 2.7E-02 | cilium organization |
| GO:0010608 | 2.7E-02 | posttranscriptional regulation of gene expression |
| GO:0003924 | 3.5E-02 | GTPase activity |
| GO:0042026 | 3.5E-02 | protein refolding |
| GO:0016772 | 3.9E-02 | transferase activity, transferring phosphorus-containing groups |
