# Supplementary material for: Life cycle adapted upstream open reading frames (uORFs) in Trypanosoma congolense: A post-transcriptional approach to accurate gene regulation
Source: PLoS One. 2018 Aug 9;13(8):e0201461. doi: 10.1371/journal.pone.0201461 (PMC6084854; doi:10.1371/journal.pone.0201461)
Supplement: S3 Table — (DOCX) [file pone.0201461.s014.docx]

**S3 Table**: Significantly enriched GO terms among genes with up regulated and consecutively down regulated uORFs from PCF to EMF and EMF to MCF, respectively, paired with decreased and increase protein translation, respectively (p<0.05).

| GO term | p-value | molecular function / biological process |
| --- | --- | --- |
| GO:0003729 | 0.02 | mRNA binding |
| GO:0004812 | 0.03 | aminoacyl-tRNA ligase activity |
| GO:0006418 | 0.03 | tRNA aminoacylation for protein translation |
| GO:0000022 | <0.05 | mitotic spindle elongation |
| GO:0030674 | <0.05 | protein binding, bridging |
| GO:0008478 | <0.05 | pyridoxal kinase activity |
| GO:0009443 | <0.05 | pyridoxal 5'-phosphate salvage |
| GO:0048037 | <0.05 | cofactor binding |
